# Computational annotation of genes differentially expressed along olive fruit development

**DOI:** 10.1186/1471-2229-9-128

**Published:** 2009-10-24

**Authors:** Giulio Galla, Gianni Barcaccia, Angelo Ramina, Silvio Collani, Fiammetta Alagna, Luciana Baldoni, Nicolò GM Cultrera, Federico Martinelli, Luca Sebastiani, Pietro Tonutti

**Affiliations:** 1 Department of Environmental Agronomy and Crop Science, University of Padova, Viale dell'Università 16, 35020 Legnaro (Padova), Italy; 2 CNR - Istituto di Genetica Vegetale - Research Division of Perugia, Via Madonna Alta 130, 06128 Perugia, Italy; 3 Scuola Superiore Sant'Anna - Pisa, Piazza Martiri della Libertà 33, 56127 Pisa, Italy

## Abstract

**Background:**

*Olea europaea *L. is a traditional tree crop of the Mediterranean basin with a worldwide economical high impact. Differently from other fruit tree species, little is known about the physiological and molecular basis of the olive fruit development and a few sequences of genes and gene products are available for olive in public databases. This study deals with the identification of large sets of differentially expressed genes in developing olive fruits and the subsequent computational annotation by means of different software.

**Results:**

mRNA from fruits of the cv. Leccino sampled at three different stages [*i.e*., initial fruit set (stage 1), completed pit hardening (stage 2) and veraison (stage 3)] was used for the identification of differentially expressed genes putatively involved in main processes along fruit development. Four subtractive hybridization libraries were constructed: forward and reverse between stage 1 and 2 (libraries A and B), and 2 and 3 (libraries C and D). All sequenced clones (1,132 in total) were analyzed through BlastX against non-redundant NCBI databases and about 60% of them showed similarity to known proteins. A total of 89 out of 642 differentially expressed unique sequences was further investigated by Real-Time PCR, showing a validation of the SSH results as high as 69%. Library-specific cDNA repertories were annotated according to the three main vocabularies of the gene ontology (GO): cellular component, biological process and molecular function. BlastX analysis, GO terms mapping and annotation analysis were performed using the Blast2GO software, a research tool designed with the main purpose of enabling GO based data mining on sequence sets for which no GO annotation is yet available. Bioinformatic analysis pointed out a significantly different distribution of the annotated sequences for each GO category, when comparing the three fruit developmental stages. The olive fruit-specific transcriptome dataset was used to query all known KEGG (Kyoto Encyclopaedia of Genes and Genomes) metabolic pathways for characterizing and positioning retrieved EST records. The integration of the olive sequence datasets within the MapMan platform for microarray analysis allowed the identification of specific biosynthetic pathways useful for the definition of key functional categories in time course analyses for gene groups.

**Conclusion:**

The bioinformatic annotation of all gene sequences was useful to shed light on metabolic pathways and transcriptional aspects related to carbohydrates, fatty acids, secondary metabolites, transcription factors and hormones as well as response to biotic and abiotic stresses throughout olive drupe development. These results represent a first step toward both functional genomics and systems biology research for understanding the gene functions and regulatory networks in olive fruit growth and ripening.

## Background

Fruit development is the result of genetically programmed processes influenced by environmental factors. To identify and characterize genes involved in these processes, different genomic approaches (ESTs, large-scale microarrays, deep transcriptome profiling, etc.) have been used in several fruit species [1] and the body of information concerning transcriptional networks and regulatory circuits involved in important physiological and developmental processes increased tremendously during the last two decades. In tomato, large-scale EST sequencing projects resulted in a better insight into molecular mechanisms of fruit ripening processes and in the identification of common transcription factors not previously associated with ripening [2,3]. Generation of ESTs and consequent discovery of genes with potential roles in fruit development have also been reported in grape berry [4,5]. In apple, an extensive analysis has been made using all EST sequences available in public databases to identify genes temporally or spatially regulated during fruit growth and development [6]. Other extensive EST sequencing projects focusing on fruit development have been set up in peach [7], melon [8] and kiwifruit [9]. Sequence information derived from advanced EST sequencing is an essential resource for functional genomics studies based on the use of microarray technology and real-time PCR. Following the pioneering work of Aharoni and co-workers [10] on strawberry, several papers have now been published on the use of microarrays in different fruit species.

*Olea europaea L*. is an evergreen species, traditionally cultivated in the Mediterranean basin. The oil that results from mechanical extraction of the fruits is a predominant component of the worldwide known 'Mediterranean diet', to which increasing attention is being paid for its health benefits and cancer-protective properties [11]. These attributes are closely related to the oil composition and to the concentration of active bio-molecules resulting from the catabolic and anabolic processes taking place throughout olive fruit development which is a long process lasting several months. The oil content of olives can reach up to 30% (fresh weight) at full ripening [12]: it accumulates in the mesocarp and, at a lower extent, in the seed [13]. Oil accumulation in the pulp increases slowly, reaching the plateau after veraison. A marked tryacylglycerol (TAG) accumulation in seed and pulp occurs after endocarp lignification, when about 40 mg of oil per fruit per week can be synthesized. The fatty acid profile of the oil accumulating in the fruit is important in relation to its nutritional properties [11]. The main fatty acid is oleic acid (C18:1), which represents about 75% of total fatty acids, followed by linoleic (C18:2), palmitic (C16:0), stearic (C18:0) and linolenic (C18:3) acid. The pattern of fatty acid synthesis and desaturation varies during maturation and ripening, according to cultivars and to environmental conditions [14,15]. Other important metabolites accumulate throughout olive fruit development. They include polyphenols [16], carotenoids [17], chlorophylls [18], sterols and terpenoids [19] all directly or indirectly affecting olive oil quality and its technological and nutritional properties.

Information concerning genetic regulation of these metabolic processes in olive is still very limited. Only few genes involved in fatty acid metabolism have been characterized [13,20-24]. A monosaccharide transporter (OeMST2), whose expression increases during fruit maturation, when a massive accumulation of sugars occurs, has been recently cloned [25]. Moreover, the gene encoding a geranylgeranyl reductase (OeCHLP) has been isolated and its role in organ development and stress response in relation to tocopherol action hypothesized [26]. Information at molecular level about polyphenol and triterpenoid metabolism is lacking, as well as the mechanisms involved in olive fruit development and ripening.

Among different strategies available for identifying differentially expressed genes, suppression subtractive hybridization (SSH) libraries have been successfully used in fruit science to elucidate mechanisms regulating anthocyanin metabolism in grape berries [27], proanthocyanidin biosynthesis in persimmon [28], processes involved in early growth and ethylene-induced ripening in banana [29,30], and in orange pigmentation [31].

This paper deals with the identification via SSH of large repertories of differentially expressed genes in developing olive fruits, and their computational annotation by means of different bioinformatic software. The identification and characterization of gene regulatory networks and key metabolic pathways during fruit growth and development represent a prerequisite for improving olive oil quality and its health-related properties.

## Results

**Figure 1:**
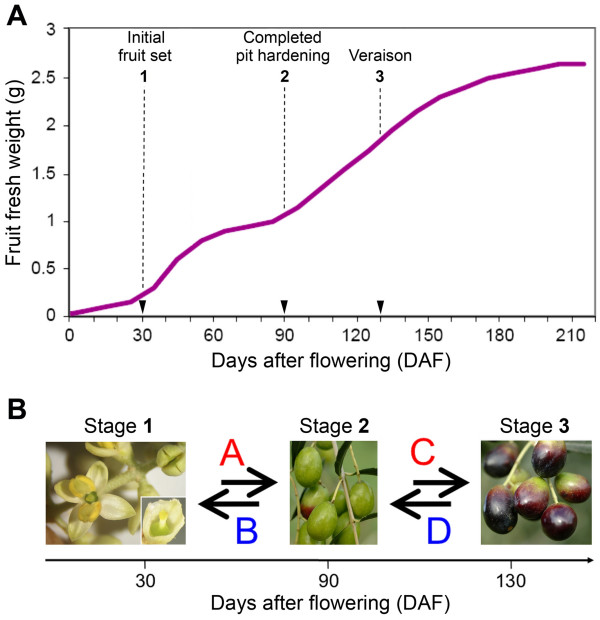
**Olive fruit growth and developmental stages considered for SSH library construction**. (A) Olive fruit (cv Leccino) growth curve expressed as fresh weight (g) accumulation. Pericarp was sampled at initial fruit set (stage 1, 30 days after flowering, DAF), end of pit hardening (stage 2, 90 DAF), and veraison (stage 3, 130 DAF). (B) Suppression subtractive hybridization (SSH) libraries were constructed by comparing samples collected at stages 1 and 2 (libraries A and B), and at stages 2 and 3 (libraries C and D). For the two forward libraries A and C, cDNA from samples collected at stages 1 and 2, respectively, were used as tester and cDNA from stages 2 and 3, respectively, were used as drivers, vice versa for the reverse subtractive libraries B and D.

The study was based on the preparation of cDNA libraries using SSH that, likewise to the differential display (DD), represents an efficient strategy to isolate genes with an antagonist expression pattern. This technique enabled to identify transcripts of genes differentially expressed among the three different developmental stages of olive fruit corresponding to initial fruit set (30 DAF), completed pit hardening (90 DAF) and veraison (130 DAF) (Figure 1).

As far as the composition of the four subtractive libraries is concerned, the number of differentially expressed sequences randomly chosen varied from a minimum of 236 to a maximum of 317 per library, with a total number of clones equal to 1,132 (Table 1). The average length of the cDNA clones was 597 bp with a wide range of variation, from 48 up to 1,283 bp. The redundancy within each single library was relatively low, ranging between 1.7% and 5.3%. Taking into account the whole set of sequences, the overall redundancy calculated among the four libraries was equal to 3.7%. The sequences of each single library were preliminarily analyzed using the CAP3 program in order to isolate the singlets and assemble contiguous and overlapping clones into contigs. This affected the comparative redundancy that increased up to 6.2%.

**Table 1:** Library characteristics defined by clone abundance and size, proportion of singlets and contigs and related redundancy.

	**Clones**				**Singlets**				**Contigs**				**Redundancy**
				
**Library**	**No.**	**%**	**Mean (bp)**	**St. Dev.**	**No.**	**%**	**Mean (bp)**	**St. Dev.**	**No.**	**%**	**Mean (bp)**	**St. Dev.**	**%**
**A**	293	25.9	522	296	139	47.4	464	313	40	52.6	767	318	3.8
**B**	317	28.0	637	328	132	41.6	583	368	35	58.4	963	207	5.3
**C**	286	25.3	694	348	114	39.9	639	383	43	60.1	835	324	4.0
**D**	236	20.8	517	320	127	53.8	506	326	64	46.2	558	356	1.7

**Total**	**1,132**	**100.0**	**597**	**332**	**500**	**42.8**	**544**	**353**	**142**	**57.2**	**747**	**349**	**6.2**

Querying with cDNA sequences the non-redundant NCBI databases allowed the attribution of a BLAST hit of 79%, 91%, 88% and 78% of the clones belonging to the A, B, C and D libraries, respectively (Table 2). The average sequence similarity was around 76%, ranging from 72% to 80%, and the median E-value for each single library ranged from 1e-47 to 1e-77.

**Table 2:** Number of hits, similarity estimates and E-values resulting from BLAST analysis of EST clones against non-redundant NCBI databases.

		**Similarity**			**E-value**
			
**Library**	**BLAST hits (%)**	**Mean**	**St.Dev.**	**Min-Max**	**Median**
**A**	79	72	16	41 - 99	1e-47
**B**	91	80	15	37 - 100	1e-71
**C**	88	73	15	45 - 100	1e-77
**D**	78	80	15	45 - 100	9e-58

The max E-value was equal to 1e--6 corresponding to the cut-off adopted for GO annotation.

**Figure 2:**
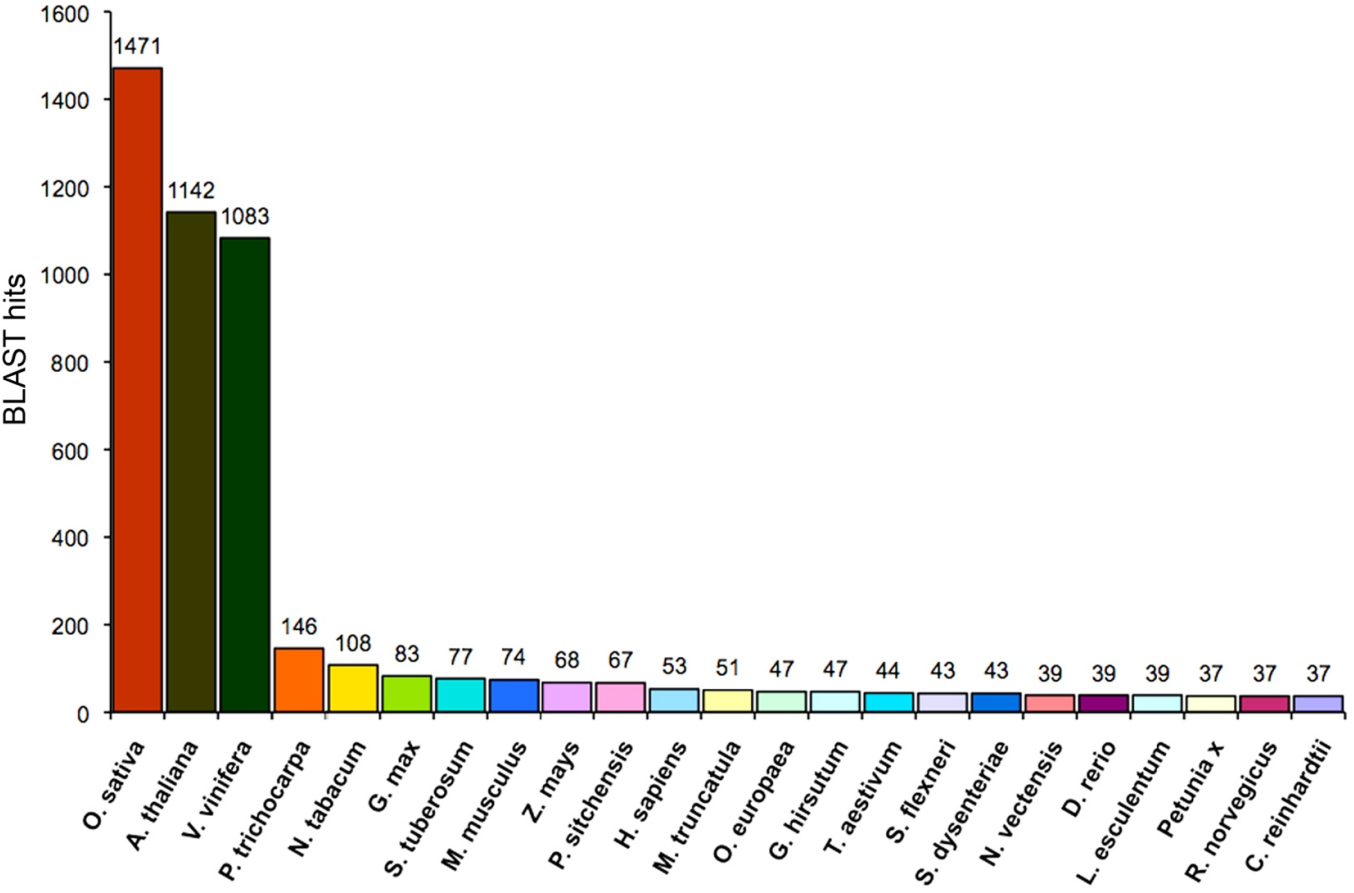
**BLAST hits as retrieved from NCBI databases**. Number of BLAST hits retrieved from NCBI databases and their distribution among different plant species and different organisms. It is worthy to note that the majority of BLAST hits were recorded for rice, Arabidopsis and grapevine, while only 47 were the entries identified for olive.

Around 75% of the BLAST hits of the olive fruit cDNA sequences were homologous to coding sequences present in the rice, *Arabidopsis *and grapevine genomes, with more than 1,000 hits per species. It is worthy to note that until now (June 2009) only 47 BLAST hits for olive could be recorded (Figure 2).

**Figure 3:**
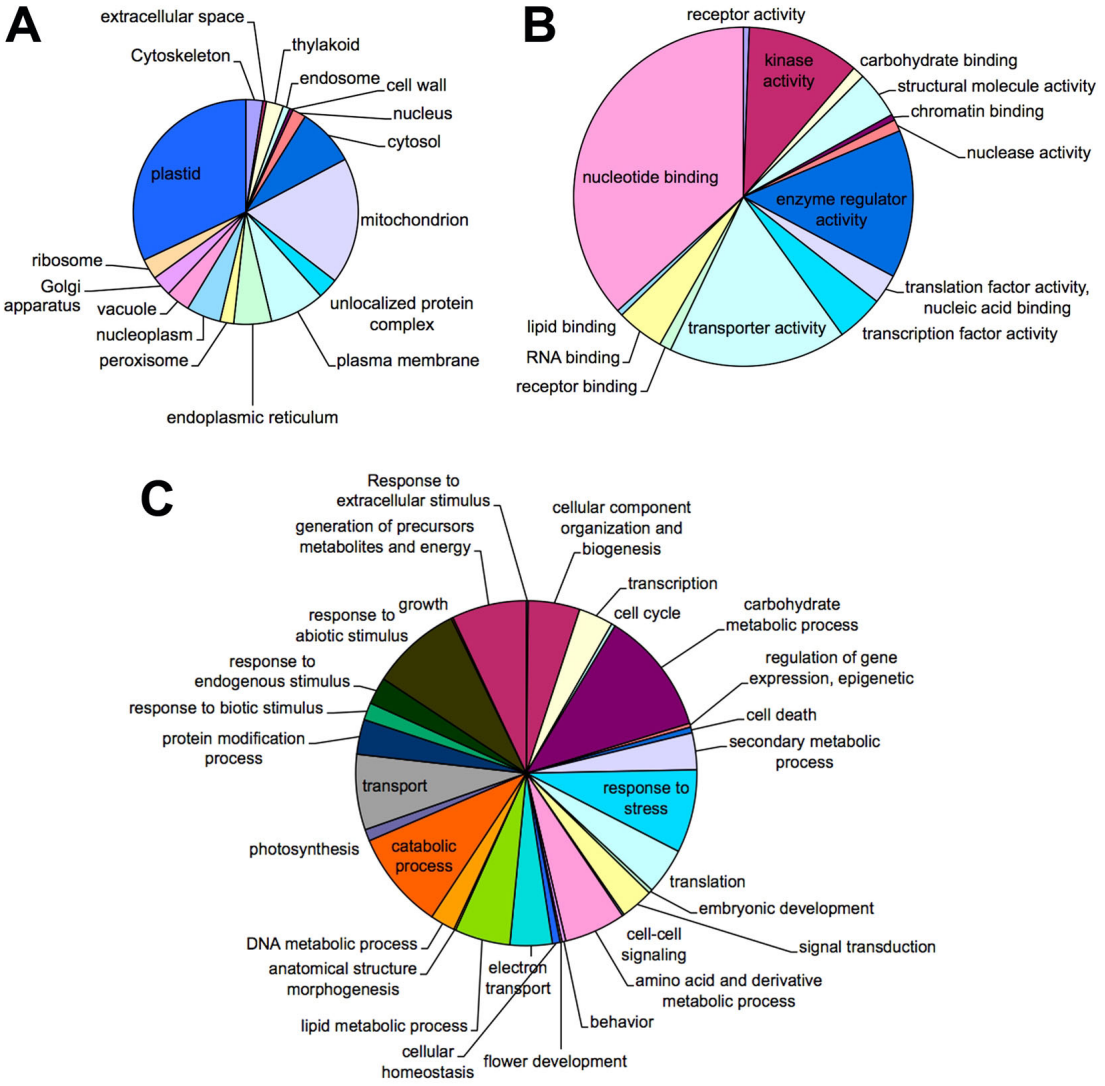
**GO terms distribution**. GO terms distribution in the cellular components (A), molecular functions (B), and biological processes (C) vocabularies. In (A) plastids and mitochondria were the most represented cellular compartments. In (B) the most represented categories were nucleotide binding proteins, followed by proteins with transport, kinase and enzymatic activities. In (C) more than 30 categories were found, being carbohydrate metabolism, response to biotic and environmental stresses, generation of precursors, metabolites and energy, and catabolic processes the most represented.

The computational analysis of the whole EST collection using the software Blast2GO allowed the annotation of the expressed sequences according to the terms of the three main Gene Ontology vocabularies, *i*.*e*. cellular compartment, molecular function and biological process (Figure 3). As far as cellular compartments are concerned, the most represented are plastids and mitochondria, with more than 50% of the total annotations, followed by cytosol, plasma membrane, endoplasmic reticulum and nucleoplasm, whereas other cellular compartments were represented at a much lower scale (Figure 3A). Concerning the molecular function, the most represented categories were those of nucleotide binding proteins, followed by proteins with transport, kinase and enzymatic activities. The other molecular functions were represented at a lower extent (Figure 3B). More than 30 categories were found for the biological process vocabulary, being carbohydrate metabolism, response to biotic and environmental stresses, generation of precursors, metabolites and energy, and catabolic processes the most represented (Figure 3C). Although numerically less represented, it is worth to mention the presence of terms related to the secondary metabolites, metabolism of lipids, synthesis of amino acids and derivatives, metabolites and their precursors, and protein modification process.

**Figure 4:**
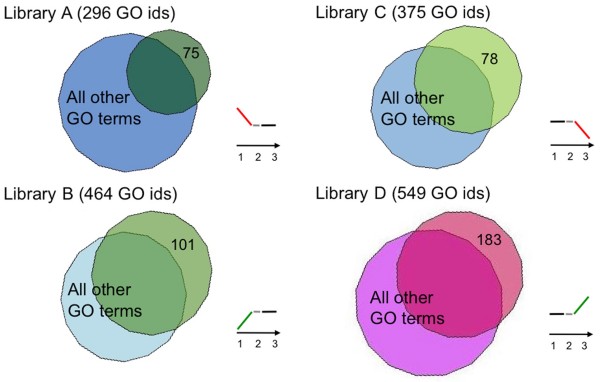
**Venn diagrams**. Diagrams showing numbers of GO terms specific of libraries A, B, C and D. Among the 296 and 464 GO terms found in the A and B libraries, 75 and 101 were associated to down- and up-regulated genes, respectively. In the C and D libraries, a total of 375 and 549 GO terms were recovered, 78 and 183 of which were related to down- and up-regulated genes, respectively.

Focusing on the GO annotation of each single subtractive library, a number of olive fruit stage-specific GO terms were identified (Figure 4). Among the 296 and 464 GO terms found in the A and B libraries, 75 and 101 were associated to down- and up-regulated genes, respectively. The most significant GO terms were encoding elements of hormone biosynthesis and signal transduction mediated by ethylene, jasmonic acid, salicylic acid, and abscisic acid, as well as biosynthesis of secondary metabolites, such as terpenoids. In the C and D libraries, a total of 375 and 549 GO terms were recovered, 78 and 183 of which were related to down- and up-regulated genes, respectively. Among these, there are GO terms associated to environmental stress responses, catabolism of secondary metabolites (as terpenes, limonene and carotene), response to hormones (gibberellins and cytokinins), and auxin signal transduction.

**Figure 5:**
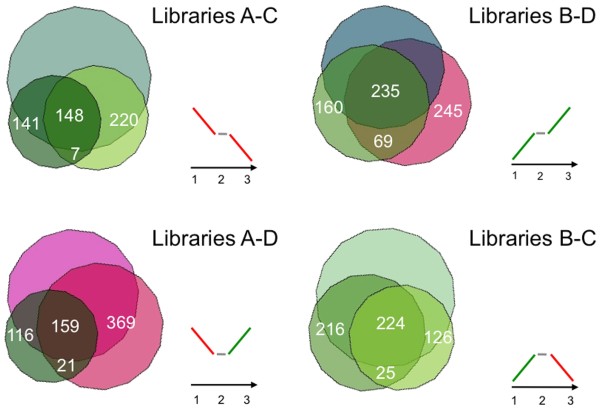
**Venn diagrams**. Diagrams showing numbers of GO terms retrieved for genes constantly or transiently down- and up-regulated throughout the three fruit developmental stages. Only 7 terms were found for the down-regulated genes, whereas as many as 69 were collected among the up-regulated ones. On the contrary, 21 and 25 terms associated to transiently up- and down-regulated genes, respectively, were found.

The analysis of GO terms shared by pair-wise library combinations allows to determine which genes are continuously or transiently down- or up-regulated during the studied process. This analysis retrieved only 7 terms for the down-regulated genes, whereas as many as 69 were those collected among the up-regulated ones. On the contrary, comparable numbers (21 vs. 25) of GO terms associated to transiently up- and down-regulated genes among the three fruit developmental stages were found (Figure 5).

**Figure 6:**
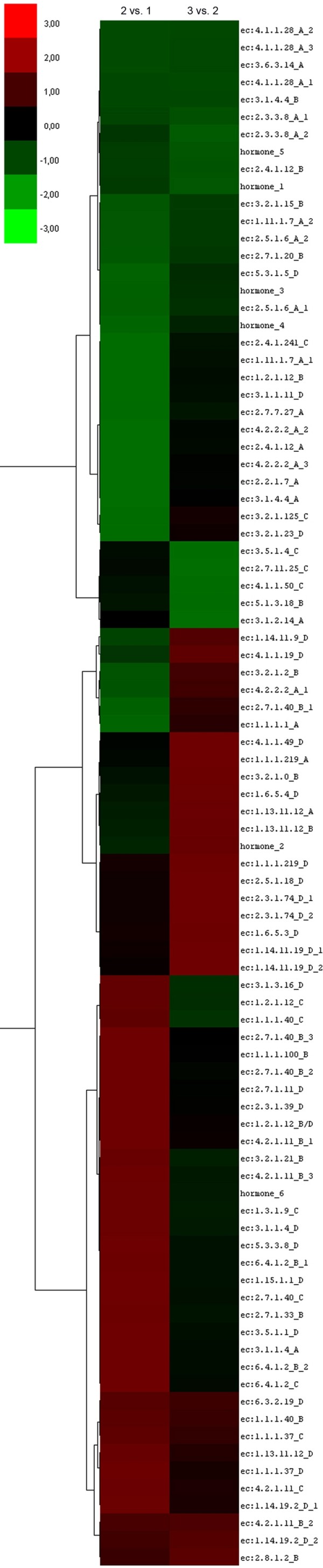
**Clustering of gene sequences performed according to the Real-Time PCR expression values**. Pair-wise analysis of expression patterns related to the selected 89 gene sequences was performed by Cluster software comparing the three different fruit stages (2 versus 1 and 3 versus 2). Different tonalities of green and red represent down- and up-regulation levels, respectively. Enzyme names corresponding to enzyme codes are listed in the supplementary materials (Additional file 2).

Quantitative Real-Time PCR experiments were carried out to corroborate the expression patterns of a subset of sequences (*i*.*e*. 89 out of 642 unisequences, equal to 14%), corresponding to 61 different genes. Expression patterns related to the selected gene sequences and estimated in pair-wise comparisons between the three different fruit stages are reported in Figure 6. The Real-Time PCR analyses validated the results from SSH experiments for 42 out of 61 genes (about 69%). The validated genes were grouped according to different expression patterns (Additional file 1).

The Kyoto Encyclopaedia of Genes and Genomes (KEGG) was queried for sequences encoding enzymes and the deduced gene products were associated to specific metabolic and/or biosynthetic pathways related to carbohydrates, fatty acid and secondary metabolism. The most represented KEGG maps associated with carbohydrate and fatty acid biosynthesis and metabolism were organized in a simple network analysis representative of major communication ways among retrieved metabolic pathways (Additional files 3 and 4). As expected, several genes encoding enzymes related to carbohydrate and fatty acid compounds were transcriptionally up- or down-regulated during olive fruit development. Several components of the carbon fixation in photosynthetic organisms (Map:00710), and starch and sucrose metabolism (Map:00500) were modulated in their expression in the early stages of fruit development (30-90 DAF). KEGG maps analysis pointed out an intense up-regulation of the majority of enzymes related to the pentose phosphate pathway (Map:00030), glycolysis and gluconeogenesis (Map:00010) along with starch and sucrose metabolism (Map:00500) (Additional file 3). Table 3 reports the enzymes involved in starch and sucrose metabolism, glycolysis and gluconeogenesis. Transcripts of enzymes involved in the synthesis of pyruvate from β-D-fructose-6-P, such as 6-phosphofructokinase (ec:2.7.1.11), glyceraldehyde-3-phosphate dehydrogenase (ec:1.2.1.12), phosphoglycerate kinase (e.c:2.7.2.3), phosphopyruvate hydratase (e.c:4.2.1.11) and pyruvate kinase (ec:2.7.1.40) were up-regulated from 30 to 90 DAF and down-regulated from 90 DAF to 130 DAF (Table 3).

**Table 3:** List of enzymes involved in carbon fixation, starch and sucrose metabolism, glycolysis and gluconeogenesis along with the library of origin of the correspondent EST clones.

**Enzyme**	**Enzyme Id**	**Library**	Metabolic pathways *								
			
			**G**	**C**	**P1**	**P2**	**F**	**S**	**Py**	**Cf**	**A**
**Alcohol dehydrogenase**	ec:1.1.1.1	A	X								
**Malate dehydrogenase**	ec:1.1.1.37	C		X					X	X	
**Malate dehydrogenase**	ec:1.1.1.40	B-C							X	X	
**Glyceraldehyde-3-phosphate dehydrogenase**	ec:1.2.1.12	B-C	X								
**Glyceraldehyde-3-phosphate dehydrogenase**	ec:1.2.1.13	D								X	
**Pyruvate dehydrogenase**	ec:1.2.4.1	B-D	X						X		
**Monodehydroascorbate reductase**	ec:1.6.5.4	D									X
**Dihydrolipoyl dehydrogenase**	ec:1.8.1.4	B	X	X					X		
**Transketolase**	ec:2.2.1.1	B-D			X					X	
**Citrate (Si)-synthase**	ec:2.3.3.1	A		X							
**ATP citrate synthase**	ec:2.3.3.8	A		X							
**Phosphorylase**	ec:2.4.1.1	A						X			
**Cellulose synthase**	ec:2.4.1.12	A						X			
**6-phosphofructokinase**	ec:2.7.1.11	B-D	X		X		X				
**Pyruvate kinase**	ec:2.7.1.40	B-C	X						X	X	
**Diphosphate-fructose-6-phosphate 1-phosphotransferase**	ec:2.7.1.90	B-D					X				
**Phosphoglycerate kinase**	ec:2.7.2.3	B	X							X	
**Glucose-1-phosphate adenylyltransferase**	ec:2.7.7.27	A						X			
**Pyruvate, phosphate dikinase**	ec:2.7.9.1	B							X	X	
**Carboxylesterase**	ec:3.1.1	D	X				X			X	
**Pectinesterase**	ec:3.1.1.11	D				X		X			
**Polygalacturonase**	ec:3.2.1.15	B				X		X			
**Beta-amylase**	ec:3.2.1.2	A						X			
**Beta-glucosidase**	ec:3.2.1.21	B						X			
**Alpha, alpha-trehalase**	ec:3.2.1.28	C						X			
**6-phospho-beta-glucosidase**	ec:3.2.1.86	C	X								
**Phosphoenolpyruvate carboxykinase**	ec:4.1.1.49	D		X					X	X	
**Phosphopyruvate hydratase**	ec:4.2.1.11	B-C	X								
**Pectate lyase**	ec:4.2.2.2	A				X					
**GDP-mannose 3,5-epimerase**	ec:5.1.3.18	B									X
**Triose-phosphate isomerase**	ec:5.3.1.1	D	X				X			X	
**Xylose isomerase**	ec:5.3.1.5	D				X	X				
**Acetyl-CoA carboxylase**	ec:6.4.1.2	B-C							X		

**Number of identified enzymes **			**11**	**5**	**2**	**4**	**5**	**8**	**8**	**10**	**2**

* G: Glycolysis and gluconeogenesis; C: Citrate cycle (TCA cycle); P1: Pentose phosphate pathway; P2: Pentose and glucuronate interconversions; F: Fructose and mannose metabolism; S: Starch and sucrose metabolism; Py: Pyruvate metabolism; Cf: Carbon fixation; A: Ascorbate and aldarate metabolism

Consistent with the accumulation of oil in the drupe that starts around pit hardening and reaches the highest rate well before ripening begins, several transcripts encoding enzymes leading to fatty acid biosynthesis (Map:00061) from glycolysis (Map:00010), through the pyruvate metabolism (Map:00620) and citrate cycle (Map:00020) were up-regulated (Additional file 4). It is worthy to mention that several enzymes were found to be up-regulated from 30 to 90 DAF, when the lipid accumulation shows an increasing rate. A similar dynamics in terms of specific transcript accumulation was recorded for several components of the FA biosynthesis as acetyl-CoA carboxylase (ec:6.4.1.2) and enoyl- [acyl-carrier-protein] reductase (ec:1.3.1.9), actively involved in the synthesis of malonylCoA and FA chain elongation, respectively (Table 4). Moreover, enzymes controlling FA chain elongation were up-regulated throughout fruit development, whereas the synthesis of short chain FAs was impaired during early development. The malonylCoA-ACP transacylase (ec:2.3.1.39), the enzyme involved in one of the early steps of FA biosynthesis, and the acyl- [acyl-carrier-protein] desaturase (ec:1.14.19.2), related to the synthesis of hexadecenoyl- [acp] and octadecenoyl- [acp], the precursors of palmitic and oleic acid, respectively, were up-regulated at veraison as well as a number of genes involved in the FA metabolism (Table 4).

**Table 4:** List of enzymes involved in fatty acid biosynthesis and metabolism along with the library of origin of the correspondent EST clones.

**Enzyme**	**Enzyme Id**	**Library**	Metabolic and biosynthetic pathway *								
			
			**B**	**M**	**G**	**Gp**	**E**	**A**	**L**	**aL**	**U**
**Alcohol dehydrogenase**	ec:1.1.1.1	A		X							
**3-oxoacyl- [acyl-carrier-protein] reductase**	ec:1.1.1.100	B	X								X
**3-hydroxyacyl-CoA dehydrogenase**	ec:1.1.1.35	D		X						X	
**Glycerol-3-phosphate dehydrogenase **	ec:1.1.1.8	B				X					
**Glycerol-3-phosphate dehydrogenase **	ec:1.1.1.94	B				X					
**Lipoxygenase**	ec:1.13.11.12	B-D							X	X	
**Unspecific monooxygenase**	ec:1.14.14.1	A-D		X				X	X		
**Acyl- [acyl-carrier-protein] desaturase**	ec:1.14.19.2	D	X								X
**Enoyl- [acyl-carrier-protein] reductase**	ec:1.3.1.9	B-C	X								
**Acyl-CoA dehydrogenase**	ec:1.3.99.3	D		X							
**[acyl-carrier-protein] S-malonyltransferase**	ec:2.3.1.39	D	X								
**Digalactosyldiacylglycerol synthase**	ec:2.4.1.241	C			X						
**Carboxylesterase**	ec:3.1.1	D			X						
**Phospholipase A2**	ec:3.1.1.4	A-D				X	X	X	X	X	
**Oleoyl- [acyl-carrier-protein] hydrolase**	ec:3.1.2.14	A	X								
**Phospholipase D**	ec:3.1.4.4	B				X	X				
**Beta-galactosidase**	ec:3.2.1.23	D			X						
**Purine nucleosidase**	ec:3.2.2	B-C					X	X			
**Enoyl-CoA hydratase**	ec:4.2.1.17	D		X						X	X
**3-hydroxybutyryl-CoA epimerase**	ec:5.1.2.3	D		X							
**Dodecenoyl-CoA isomerase**	ec:5.3.3.8	D		X							
**Acetyl-CoA carboxylase**	ec:6.4.1.2	B-C	X								

**Number of identified enzymes**			**6**	**7**	**3**	**4**	**3**	**3**	**3**	**4**	**3**

* B: Fatty acid biosynthesis; M: Fatty acid metabolism; G: Glycerolipid metabolism; Gp: Glycerophospholipid metabolism; E: Ether lipid metabolism; A: Arachidonic acid metabolism; L: Linoleic acid metabolism; aL: Alpha-Linolenic acid metabolism; U: Biosynthesis of unsaturated fatty acids.

**Figure 7:**
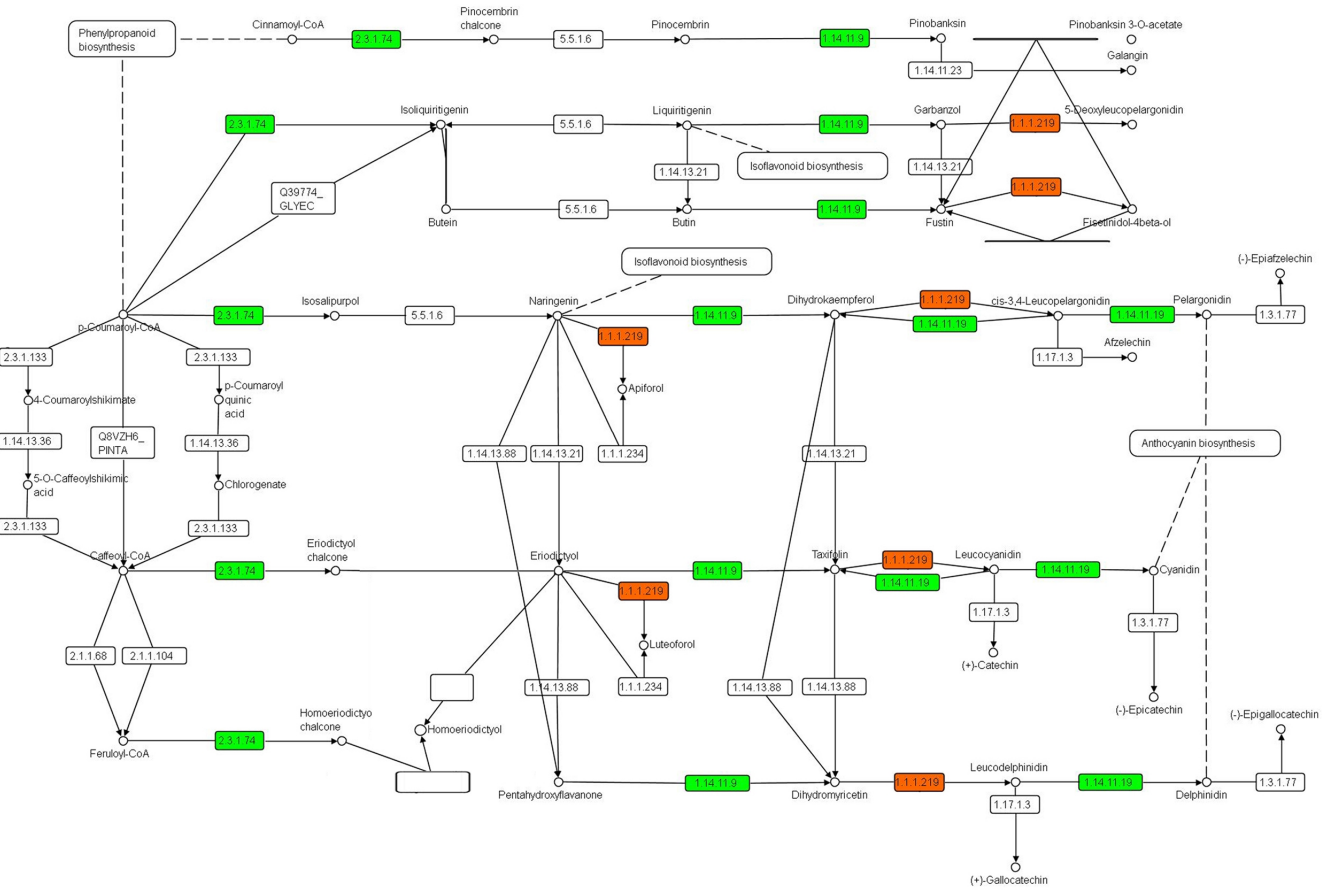
**KEGG pathway for flavonoid biosynthesis (Map:00941)**. The isoforms of four different enzymes (*i.e*. dihydrokaempferol 4-reductase, ec:1.1.1.219; flavanone 3-dioxygenase, ec:1.14.11.9; naringenin-chalcone synthase, ec:2.3.1.74; and leucocyanidin oxygenase, ec:1.14.11.19) controlling flavone and flavonol as well as anthocyanin biosynthesis were mapped (see also Additional file 5).

KEGG maps were also produced for secondary metabolic pathways. Considering the flavonoids as an example, the isoforms of four different enzymes (*i*.*e*. dihydrokaempferol 4-reductase, flavanone 3-dioxygenase, naringenin-chalcone synthase, and leucocyanidin oxygenase) controlling flavone and flavonol as well as anthocyanin biosynthesis were mapped (Figure 7). Transcripts encoding these enzymes were more abundant during the transition from 90 to 130 DAF, thus suggesting an increased accumulation of the related metabolites during late fruit development. A transient down-regulation of the dihydrokaempferol 4-reductase gene (ec:1.1.1.219) was recorded at 90 DAF (Additional file 5). Two isoforms of a peroxidase specifically involved in four different steps of the phenylpropanoid biosynthesis as well as few enzymes involved in alkaloid biosynthesis (Map:00950; Map:00960), limonene and pinene degradation (Map:00903) and caffeine metabolism (Map:00232) were also identified (Additional file 5).

**Figure 8:**
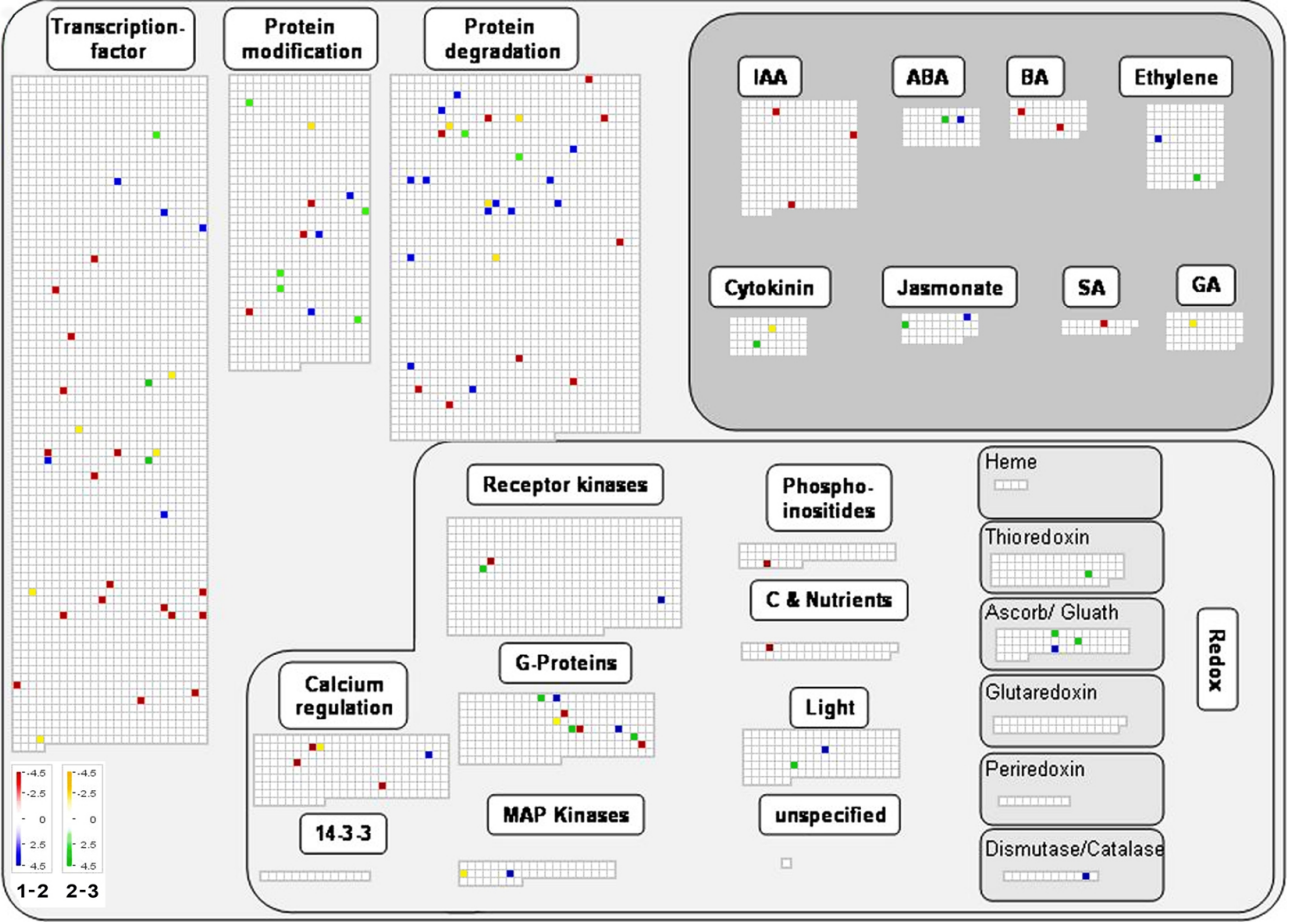
**Regulatory network map constructed with MapMan software using the olive fruit EST dataset**. Several genes encoding transcription factors were mainly down-regulated throughout fruit development, while some others were related to protein modification and degradation. Genes related to the biosynthesis and action of IAA, ABA, GA, ethylene, cytokinins, JAs and SA appeared to be differently regulated according to the type of hormone.

The analysis of the olive EST dataset with the MapMan software enabled to reconstruct overview metabolism maps (Additional file 6) and to group sequences in the main regulatory networks (Figure 8). As far as the *in silico *expression analysis of all olive EST clones linked to the Arabidopsis genechip sequences (Affymetrix), most genes proved to be associated to constantly and transiently regulated genes during fruit development involved in cell wall synthesis and breakdown, fatty acid biosynthesis and lipid breakdown, starch and sucrose metabolism, glycolysis, and secondary metabolism (*i*.*e*., terpenoids and flavonoids). Gene products involved in amino acid biosynthesis and metabolism were also identified (Additional file 6). Furthermore, several genes encoding transcription factors were mainly down-regulated throughout fruit development, while some others were related to protein modification and degradation (Figure 8). Genes related to hormone biosynthesis and action appeared to be differently regulated according to the type of hormone. A down-regulation of genes involved in auxin biosynthesis and metabolism, as an oxido/reductase and an IAA-amino acid synthase, occurred between 30 and 90 DAF, while during late development the expression of auxin responsive factors such as ARF1 and ARF7, were up- and down-regulated, respectively. By contrast, the synthesis of abscisic acid (ABA) was clearly stimulated throughout development, since two key enzymes encoded by *ABA2 *(ABA DEFICIENT 2) and *AAO3 *(ABSCISIC ALDEHYDE OXIDASE 3), were up-regulated at completed pit-hardening and veraison, respectively. In addition, a down-regulation of a gene encoding a GA-regulated protein occurred between completed pit hardening and veraison. During the same developmental phase, an up-regulation of the ARR1, a protein involved in the citokinin response, as well as a down-regulation of an UGT2, encoding a UDP-glucosyl transferase, actively involved in the metabolism of the hormone, were observed. Genes involved in jasmonic acid (JA) metabolism were up-regulated throughout fruit development. A transcriptional up-regulation of an ethylene receptor of the ERS type as well as a down-regulation of genes involved in brassinosteroid (BR) biosynthesis and action were observed during early development.

Taking into account genes related to biotic and abiotic stress responses, dual multiple contingency tests were performed to identify GO terms associated to sequences significantly and antagonistically distributed between libraries. The results of this analysis evidenced the abundance of sequences related to light stimulus (GO:0009416) and radiation (GO:0009314), as well as to biotic and abiotic stresses (Table 5). Sequences related to light stimulus and radiation resulted to be up-regulated from 30 to 60 DAF, while those associated with response to oxidative stresses were down-regulated during the same developmental period.

**Table 5:** Dual multiple contingency tests performed to identify GO terms associated to sequences significantly and antagonistically distributed between libraries for genes related to biotic and abiotic stress responses.

**Stages**	**GO Id**	**GO term**	**χ^2^**	**P value**	**Stage 1**	**Stage 2**	**Stage 3**
1-2	GO:0006979	Response to oxidative stress	20.9	1.59e-06	18	7	-
1-2	GO:0009266	Response to temperature stimulus	10.8	1.13e-03	5	0	-
1-2	GO:0009314	Response to radiation	4.3	1.75e-02	0	16	-
2-3	GO:0009314	Response to radiation	5.0	1.72e-02	-	12	3
1-2	GO:0009408	Response to heat	5.2	1.71e-02	3	0	-
1-2	GO:0009416	Response to light stimulus	4.3	1.75e-02	0	16	-
2-3	GO:0009416	Response to light stimulus	6.6	5.50e-03	-	12	2
2-3	GO:0009615	Response to virus	4.6	1.18e-02	-	6	0
2-3	GO:0009617	Response to bacteria	5.7	5.80e-03	-	7	0
1-2	GO:0009624	Response to insects	26.8	8.17e-07	12	1	-
2-3	GO:0009644	Response high light intensity	4.6	1.18e-02	-	6	0
1-2	GO:0042221	Response to chemical stimulus	12.1	6.67e-04	20	19	-
1-2	GO:0051707	Response to other organisms	20.9	1.59e-06	16	7	-
2-3	GO:0051707	Response to other organisms	5.7	5.60e-03	-	7	0

## Discussion

*Olea europaea *L. is a common tree species of the Mediterranean basin that plays a peculiar role in the landscape characterization and represents a major agricultural commodity as source of olive oil. Olives are not only a significant food source, but also contribute to human health and are becoming popular in health-conscious diets far beyond the Mediterranean area of olive oil traditional use. Taking into account the increasing worldwide commercial interest of olive oil and the lack of information on its genomic features, a functional genomic approach, able to gain insights into the genetic and molecular aspects controlling fruit development and ripening, may be considered of primary interest.

A number of physiological and biochemical data is available on growth, development and ripening of the olive drupe but, unlike other fruit species such as peach, apple and grape, information on olive gene sequences and gene products is very limited in the main public gene databases. This study was carried out on cv. Leccino, one of the most widespread Italian varieties, characterized by a relatively short fruit developmental cycle, and a high degree of synchronization of processes defining ripening.

The SSH approach allowed the identification of 1,132 differentially expressed gene sequences in three selected developmental stages of the olive fruit, named initial fruit set (30 DAF), completed pit hardening (90 DAF) and veraison (130 DAF). All the sequences were deposited in the NCBI databases on August 28, 2008 [GenBank: FL683310-FL684411, ESTdb: 60119979-60121080]. This contribution represented a major implementation of the public olive EST repertories that currently include a total number of 3,859 sequences (last verified on July 27, 2009).

The SSH technique confirmed its suitability for transcriptome studies and for identifying genes differentially expressed along fruit development in olive. In fact, of the 1,132 differentially expressed sequences as many as 642 demonstrated to be unique sequences. Among these, 89 (14%) corresponding to 61 different key genes, further investigated by quantitative Real-Time PCR, pointed out a validation of SSH findings as high as 69%

Olive fruits showed significant differences for the distribution of GO terms among ontological vocabularies and categories in relation to the developmental stages. GO terms involved in carbohydrate, fatty acid and flavonoid metabolism were analyzed by setting up KEGG maps. As far as carbohydrates are concerned, genes involved in carbohydrate metabolism were modulated in their expression and, in particular, the expression pattern of genes related to starch metabolism was coherent with a temporary role of starch as storage compound during fruit early development. Sequences of enzymes involved within the pentose phosphate pathway, glycolysis and gluconeogenesis, along with starch and sucrose metabolism, were up-regulated. The up-regulation of genes encoding enzymes involved in the synthesis of pyruvate from different substrates occurring during early development, may indicate that the fruit at this stage is highly energy demanding. There are two sources of assimilates for fruit growth in olive. The major source is certainly the sugars translocated in the phloem from leaves or sites of storage, comprising mannitol, raffinose, stachyose, and sucrose [32]. The secondary source is sugars formed by photosynthesis in developing fruits themselves that remain green for a considerable period and retain active chlorophyll even when they change colour as approaching maturity. While chlorophyll is mostly in the exocarp, the mesocarp has been shown to contain significant amounts of phosphoenol pyruvate [12], the CO_2 _fixation enzyme of the CAM and C4 photosynthetic pathway.

Genes related to fatty acid (FA) biosynthesis appeared to be up-regulated throughout development although transcripts of specific enzymes accumulate at different extent depending on the developmental stage. Enzymes controlling FA chain elongation were up-regulated throughout fruit development: the synthesis of short chain FAs was impaired during early development whereas the synthesis of the precursors of palmitoleic and oleic acids was up-regulated during late development. This finding is consistent with the oil accumulation pattern in olive mesocarp, that starts around 40-60 DAF and reaches at ripening the highest amount, confirming what previously observed [20,21]. A number of genes involved in the FA metabolism appeared to be differentially expressed throughout development with a significant up-regulation at veraison. This might be interpreted as a homeostatic reaction to the large FA accumulation occurring at this developmental stage.

Taking into account the secondary metabolites, genes involved in phenylpropanoid and alkaloid biosynthesis and caffeine, limonene and pinene metabolism appeared to be differentially expressed throughout fruit development. Dihydrokaempferol 4-reductase, flavanone 3-dioxygenase, naringenin-chalcone synthase, and leucocyanidin oxygenase, four enzymes controlling flavone and flavonol, as well as anthocyanin biosynthesis, were up-regulated from 90 to 130 DAF, thus suggesting an increased accumulation of the related metabolites during late development. Flavonoids are important secondary metabolites precursor of flavonols and anthocyanins, the latter being responsible of the color development occurring at ripening. Key genes related to the anthocyanin biosynthetic pathway (chalcone synthase, CHS, flavanone 3-hydroxylase, F3H, dihydroflavonol reductase, DFR, and anthocyanidin synthase, ANS) proved to be up-regulated at veraison stage. A similar coordinated up-regulation of these genes has been observed in grape berries at the onset of ripening, at the time of pigmentation changes [33].

The relatively high abundance of transcripts related to hormones supports their key regulatory role in olive fruit development, as demonstrated in several other fruits [34]. The synthesis of ABA is clearly stimulated throughout fruit development, as demonstrated by the up-regulation of two key enzymes of its biosynthetic pathway. The pattern of expression changed according to the type of hormone. The observed down-regulation of genes involved in auxin biosynthesis and metabolism is consistent with a lowering of the auxin content reported in other fruits throughout development. The different regulation of auxin responsive factors, such as ARF1 and ARF7, which act as negative and positive regulators of IAA responsive genes, respectively [35], might imply a decreased sensitivity to the hormone during late fruit development. This is consistent with a negative regulation of auxin on the onset of the ripening syndrome observed in non climacteric fruits. At late developmental stage, a down regulation of the zeatin O-glucosyltransferase 2, actively involved in CK metabolism, as well as an up-regulation of ARR1, a protein involved in the CK response, have been observed. ARR1 is a type-B ARR transcription factor involved in CK-responsive phenomena. It has been proposed that ARR1, together with ARR10 and ARR12, redundantly play pivotal roles in the AHK-dependent phosphorelay signaling in response to CK [36]. Taking into account that CK metabolic enzymes are up-regulated by the hormone, these transcriptional changes may reflect a lowering of CK concentration, along with an increase of fruit sensitivity to CK occurring during late development. The veraison is a developmental stage characterized by a strengthening of the fruit sink action, that, also in olive, might be regulated by a complex hormone cross-talk and interaction, as demonstrated in other non climacteric fruits as grape berries [37]. In spite of any signal variation in terms of expression of genes related to ethylene biosynthesis, an up-regulation of an ERS type ethylene receptor has been observed during early fruit development. This might imply an increase in sensitivity of the fruitlet to ethylene, the hormone involved in the regulation of the immature fruit physiological drop that in olive occurs between fruit set and pit hardening [38]. Genes involved in the jasmonates (JAs) metabolism and brassinosteroids (BRs) biosynthesis were up- and down regulated, respectively. If the gene expression pattern is mirrored by a similar evolution of JA and BR concentration in fruit tissues, this would indicate that the role of JAs and BRs in olive is different from other fruit types such as peach and grape. In fact, in peach it has been demonstrated that JAs delay fruit ripening [39], while BRs stimulate the onset of veraison in grape berry [40].

The dual multiple contingency tests allowed the identification of GO terms related to biotic and abiotic stress responses significantly and antagonistically distributed between libraries. The transcriptional profile of genes related to light, temperature and biotic stresses parallels the dynamics of day length and light intensity, both peaking at the pit hardening stage. Moreover, these data may indicate that genes responding to environmental stimuli are transcriptionally regulated up to pit hardening, and post-transcriptionally up to the veraison.

## Conclusion

As a concluding remark, the SSH technique allowed to identify a set of 642 differentially expressed unique sequences. Among these, 89 (14%) corresponding to 61 different key genes were further investigated by Real-Time PCR, pointing out a validation of the SSH results as high as 69%. The bioinformatic annotation of all gene sequences was useful to shed light on metabolic pathways and to understand specific regulatory networks. In fact, data here reported represent a significant contribution to the elucidation of transcriptional aspects related to carbohydrates, FAs, secondary metabolites, transcription factors and hormones as well as response to biotic and abiotic stresses. Particularly interesting are data related to hormones, pointing out the complexity of the role played by these compounds in olive fruit development and ripening.

These molecular and bioinformatic data represent a first step toward both functional genomics and systems biology research for understanding the gene functions and regulatory networks in olive fruit growth, development and ripening.

## Methods

### Plant material

In an orchard located nearby Perugia (Italy), growth of olive (*Olea europaea *L., cv Leccino) fruits was determined by monitoring fresh weight accumulation. Olive samples were collected at 30 days after flowering (DAF) (initial fruit set, stage 1), 90 DAF (completed pit hardening, stage 2) and 130 DAF (veraison, stage 3)] (Figure 1).

### RNA extraction

Total RNA was extracted from pericarp of about 16 fruits for each sampling date, using the RNeasy Plant Mini Kit (Qiagen). Contaminating genomic DNA was removed from total RNA by two DNase treatments. The first was performed using the RNase-Free-DNase Set (Qiagen) during RNA isolation procedure. After sample elution, total RNA was treated with DNAse I (Promega). The RNA aqueous method (Ambion) was applied to purify total RNA from phenolic compounds and other substances that could inhibit reverse transcription. The RNA was quantified by both, spectrophotometer and gel electrophoresis on a denaturing agarose gel. The mRNA was isolated from about 400 μg of total RNA for each sample using oligo dT cellulose resin.

### cDNA synthesis and library construction

For all samples, the first strand cDNA was synthesized with reverse transcriptase using oligo dT primer, modified for a tail containing *Rsa*I restriction site. The second strand was synthesized by incubating the first strand cDNA with DNA polymerase I, RNAse H and DNA ligase at 16°C for 2.5 hrs. T4 polymerase was added to blunt the ends of the cDNA to facilitate the ligation of adapters in the later steps. The samples were extracted with phenol/chloroform. Subtracted and reverse subtracted cDNA libraries were constructed using a combination of a PCR-based subtraction kit (Clontech) and a unique subtraction procedure (Rx Biosciences). Four subtracted cDNA libraries have been constructed to compare samples collected at 30 and 90 DAF (libraries A and B) and samples at 90 and 130 DAF (libraries C and D) (Figure 1). For the two forward libraries A and C, cDNA from samples collected at 30 and 90 DAF, respectively, were used as tester and cDNA from 90 and 130 DAF, respectively, were used as drivers, vice versa for the reverse subtractive libraries B and D. For DNA isolation alkaline lyses method was followed. The clones were grown in 0.5 ml of Terrific Broth (TB) overnight in 96 well plates. An aliquot was saved for glycerol stocks. Rest culture was centrifuged in HT-6000BSorvall centrifuge and re-suspended in P-1 buffer. Following re-suspension, buffer P-2 was added to lyse the cells. After 2 min, buffer P3 was added to neutralize the solutions. The lysate was transferred to 96-well filter plates then the centrifugation was performed at 2,000 g for 2 min. The clear lysate thus obtained was mixed with 0.7 volumes of ethanol and centrifuged at 6,500 g in HT-6000B centrifuge for 20 min. The pellet was once washed with 70% ethanol, dried dissolved and re-suspended in 50 μL of sterile water. The complete analysis was performed using CLC Combined Workbench 3 software. The sequence data were imported in the directory files of the software. The software has data bank of various vectors in use for library construction, if not it was imported from Manufacture's web site (Invitrogen). The sequence data were mass aligned with the vector and the sequences homologous to vector were mass trimmed. The vector trimmed sequences were mass blasted in batches using CLC software. The resulting files were saved and exported into Microsoft excel format. The sequencing was performed using ABI3700 automatic DNA sequencers using 25-50 ng of DNA template according to manufactures protocol. These experimental steps have been performed at Rx Biosciences Lab (Rockville, MD, USA).

### Gene Ontology annotation

Nucleotide sequences retrieved by SSH were screened for vector contamination by using a home made BioPearl script. Once cleaned by vector residuals, all sequences were used for contig assembly by using a web interface of the CAP3 software (CAP3, , [41]) and redundancy within and among libraries were calculated as ratio of sequences belonging to a contig out of the total sequences considered. Computational annotation of the four olive EST datasets was performed using the Blast2GO software v1.3.3 (, [42,43] as described in [44] with minor modifications. Briefly, a sequence length threshold of 200 bp was considered for all libraries and used to split the datasets in according to the length. BlastX algorithm was used for both short and long sequence datasets with different parameters according to the length. Blast expectation value threshold was constantly set to 10, whereas HSP length cutoff was set at 15 and 33, respectively. Similarly, the Blast2GO software v1.3.3 was used to obtain GO information from retrieved database matches. Annotation of all sequences was performed by using default parameters on the two ranges of length previously described. Furthermore, InterPro Scan [45] was performed to find functional motifs and related GO terms by using the specific tool implemented in the Blast2GO software with the default parameters. Finally, the 'Augment Annotation by ANNEX' function was used to refine annotations (, [46]). The GOslim 'goslim_plant.obo' was used to achieve specific GO terms by means of a plant-specific reduced version of the Gene Ontology .

Annotation distribution among originated libraries was represented by Venn diagrams by computing all retrieved annotation with the VennMaster software with the default parameters .

Enzyme mapping of annotated sequences was done by direct GO to Enzyme annotation and used to query the Kyoto Encyclopaedia of Genes and Genomes (KEGG - , [47-49]) to define the main metabolic pathways involved.

MapMan  analysis was done using the olive dataset properly rearranged as input files. The *Arabidopsis *proteome was downloaded from  and the olive transcriptome dataset used as query for local BLASTX analysis. Once blasted, the Arabidopsis AGI code relative to all BLAST hits with an E-value equal or greater than E-6 were recovered by means of an home made Perl script. Retrieved AGI codes were then converted in ATH1-121501 genechip identifiers (Affymetrix) by using the PhyloGenie web interface ; [50]). For each library, an arbitrary expression values were assigned to all EST-linked Affymetrix identifiers, and the originated dataset used as input form for subsequent MapMan analysis. Finally, a time course representation of whole olive EST dataset related to primary and secondary metabolites and cellular processes environment was done by mapping the datasets within the appropriate MapMan pathways.

### Real-Time PCR analysis

Quantitative Real-Time PCR experiments were carried out to validate some of the genes isolated by SSH and characterized by GO. Among the whole dataset of non-redundant sequences, 89 gene sequences belonging to key biosynthetic and metabolic pathways were selected according to the length (over 100 bp) and e-value (higher than 1E-6). All cDNAs were prepared from fruits collected at three developmental stages (30, 90, and 120 DAF), corresponding to the ones used for the construction of SSH libraries, using the Super Script Reverse Transcriptase kit (Invitrogen).

Specific primer pairs for each of the sequences were designed (Additional file 7) and tested for their activity at 60°C by conventional PCR. Different primers were also designed to discriminate putative isoforms. Quantitative Real-Time RT-PCR analyses were then performed using a thermal cycler 7300 Real-Time PCR System (Applied Biosystem) equipped with a 96 well plates system with the SYBR green PCR Master Mix reagent (Applied Biosystem). All Real-Time PCR experiments were performed with two independent sets of RNA samples: each analysis was performed in a final volume of 20 μl containing 2 μl of cDNA diluted 1:50, 0,3 μM of each primer, and 10 μl of 2× SYBR Green PCR Master Mix according to the manufacturer's instructions. The following thermal cycling profile was used for all PCRs: 95°C for 20 sec, 50 cycles of 95°C for 10 s and 60°C for 1 min. All quantifications were normalized to the *Olea europaea *Elongation Factor 1 gene used as housekeeping gene and amplified in the same conditions.

Data resulting from quantitative Real-Time PCR were corrected on the basis of the housekeeping gene by the ΔΔCt method. Pair-wise analyses of gene expression values at the three developmental stages were performed by comparing fruit stages (2 versus 1 and 3 versus 2). These two series of ratios were treated by Cluster 3.0 software [51]. All data were normalized and transformed in a logarithmic scale to compare expression levels of all genes and group them according to expression patterns.

## Authors' contributions

PT, LB, LS, and AR conceived the research plan. FA, FM and NGMC collected samples and carried out RNA extractions. SC performed Real-Time PCR experiments. GG, GB, FM, and FA, performed computational analyses. GG, and GB developed and implemented the annotation and bioinformatics analyses. AR, GB, PT; and LB drafted the manuscript. All authors read and approved the manuscript.

## Supplementary Material

Additional file 1 **RT-PCR validation**. Results of the validation of 42 genes analyzed by quantitative Real-Time PCR and grouped according to eight different subgroups of expression patterns among the three fruit developmental stages. Click here for file

Additional file 2 **List of enzyme names and codes**. List of enzyme names, codes and library of the 89 gene sequences tested by quantitative Real-Time PCR. Click here for file

Additional file 3 **KEGG map**. KEGG pathway for starch and sucrose metabolism (Map:00500) (A) combined with glycolysis and gluconeogenesis (Map:00010) (B). Box colors: blue and green correspond to up-regulated genes between olive stages 1 and 2, and 2 and 3, respectively, whereas red and yellow correspond to down-regulated genes between olive stages 1 and 2, and 2 and 3, respectively; gray indicates transiently regulated genes from stage 1 to stage 3. Click here for file

Additional file 4 **KEGG map**. KEGG pathway related to fatty acid biosynthesis (Map:00061) (A) and metabolism (Map:00071) (B). Box colors: blue and green correspond to up-regulated genes between olive stages 1 and 2, and 2 and 3, respectively, whereas red and yellow correspond to down-regulated genes between olive stages 1 and 2, and 2 and 3, respectively; gray indicates transiently down-regulated genes while violet indicates constantly and down-regulated genes from stage 1 to stage 3. Click here for file

Additional file 5 **Secondary metabolism enzymes**. List of enzymes involved in the biosynthesis and metabolism of secondary metabolites, such as steroids, phenylpropanoids, flavonoids, alkaloids and specific products like caffeine, limonene and pinene. Click here for file

Additional file 6 **MAPMAN map**. Overview MAPMAN metabolism map showing several constantly and transiently up- and down-regulated genes related to cell wall synthesis and breakdown, fatty acid biosynthesis and lipid breakdown, starch and sucrose metabolism, glycolysis, Calvin cycle and the secondary metabolism (*e*.*g*., terpenoids, flavonoids, phenols). Signal colors: blue and green correspond to up-regulated genes between olive stages 1 and 2, and 2 and 3, respectively, whereas red and yellow correspond to down-regulated genes between olive stages 1 and 2, and 2 and 3, respectively; cyan and violet indicate, respectively, constantly up-regulated and down-regulated genes from olive stage 1 to stage 3; gray and orange indicate, respectively, transiently down- and up-regulated genes. Click here for file

Additional file 7 **List of specific primers used for RT-PCR analysis**. List of specific primers designed on the selected key genes for the validation of their expression patterns by Real-Time PCR analysis. Click here for file
