# Supplementary figures and images for: Computational annotation of genes differentially expressed along olive fruit development
Source: BMC Plant Biol. 2009 Oct 24;9:128. doi: 10.1186/1471-2229-9-128 (PMC2774695; doi:10.1186/1471-2229-9-128)

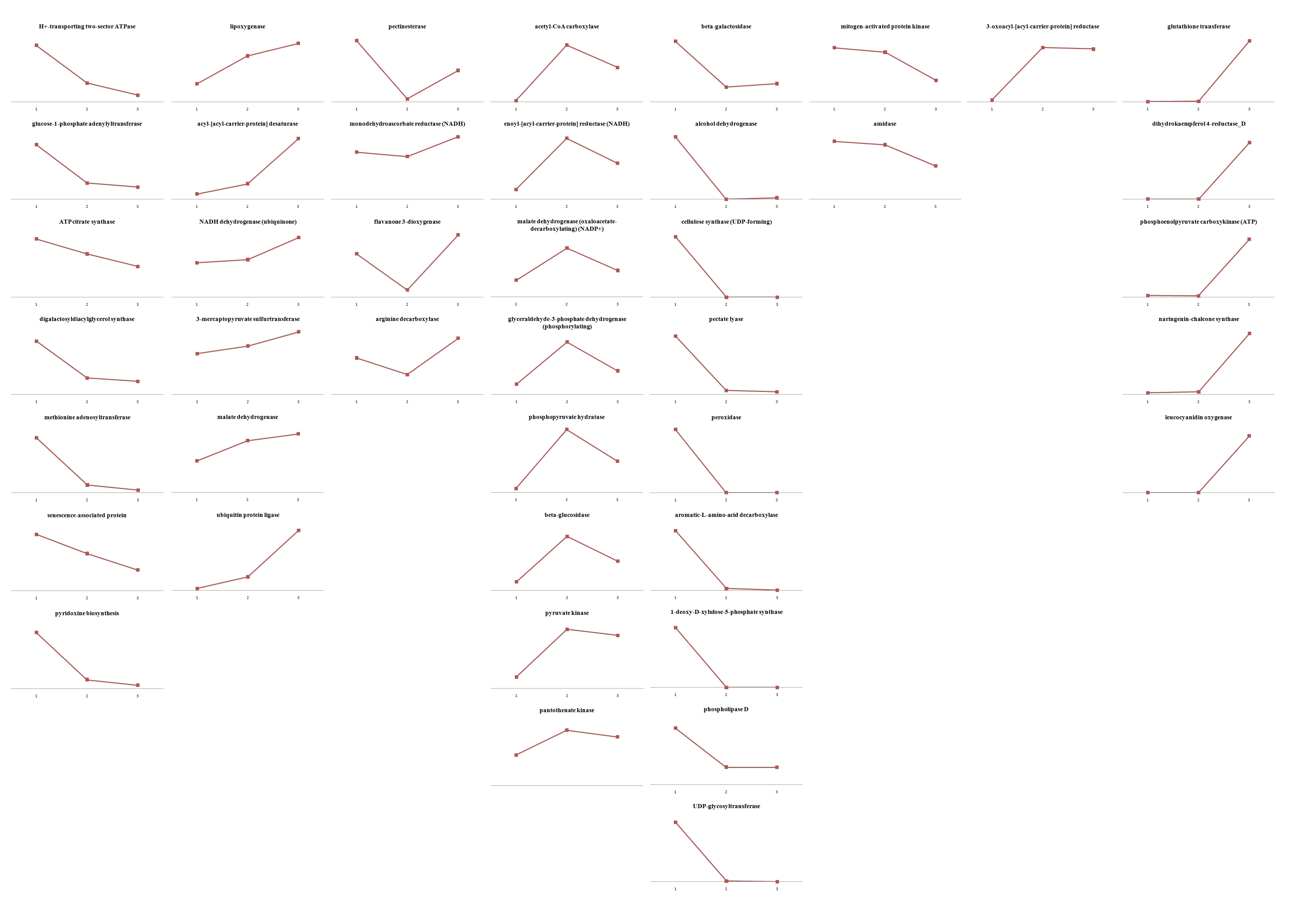

Supplement: Additional file 1 — RT-PCR validation. Results of the validation of 42 genes analyzed by quantitative Real-Time PCR and grouped according to eight different subgroups of expression patterns among the three fruit developmental stages. [file 1471-2229-9-128-S1.JPEG]

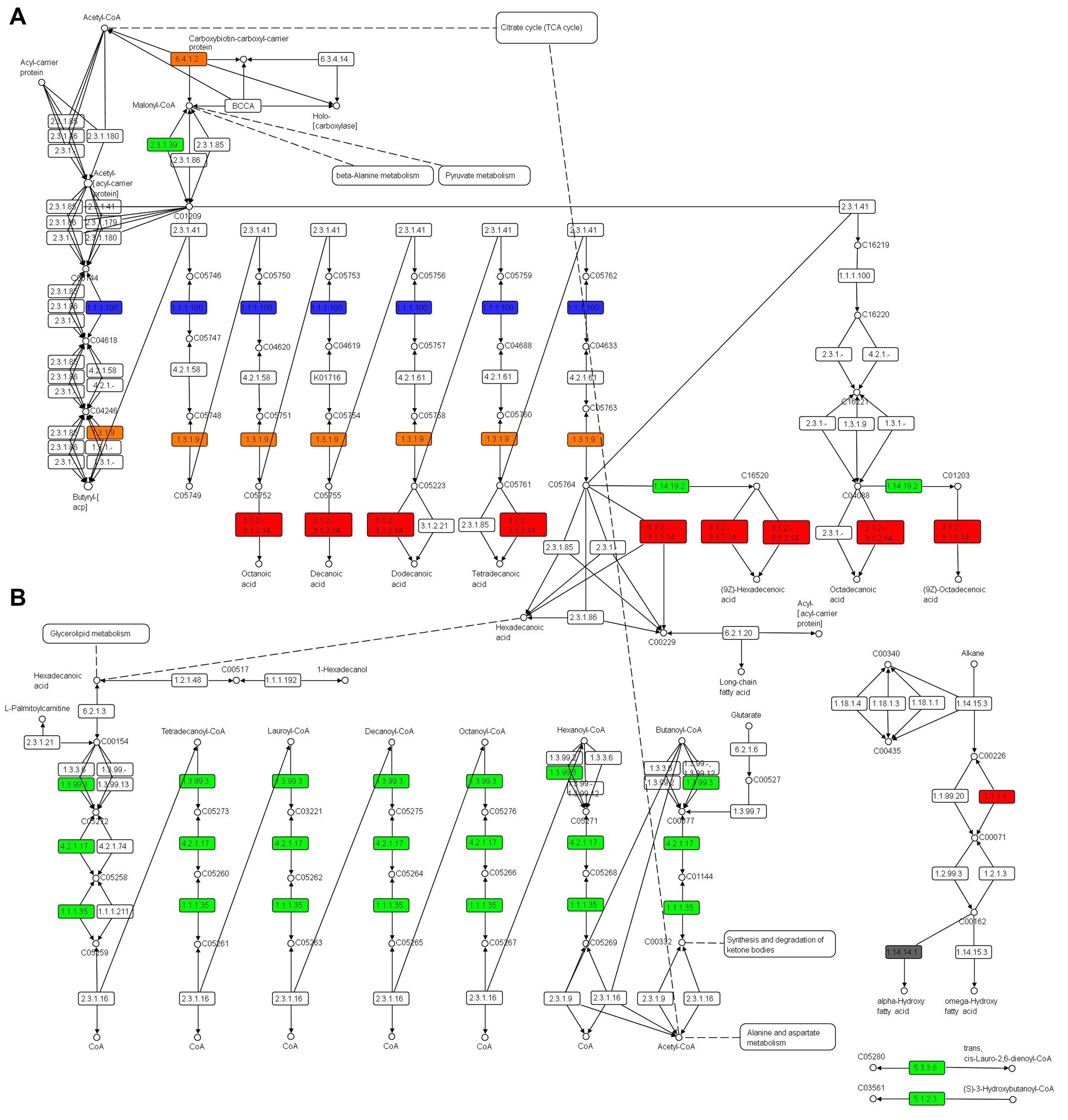

Supplement: Additional file 3 — KEGG map. KEGG pathway for starch and sucrose metabolism (Map:00500) (A) combined with glycolysis and gluconeogenesis (Map:00010) (B). Box colors: blue and green correspond to up-regulated genes between olive stages 1 and 2, and 2 and 3, respectively, whereas red and yellow correspond to down-regulated genes between olive stages 1 and 2, and 2 and 3, respectively; gray indicates transiently regulated genes from stage 1 to stage 3. [file 1471-2229-9-128-S3.JPEG]

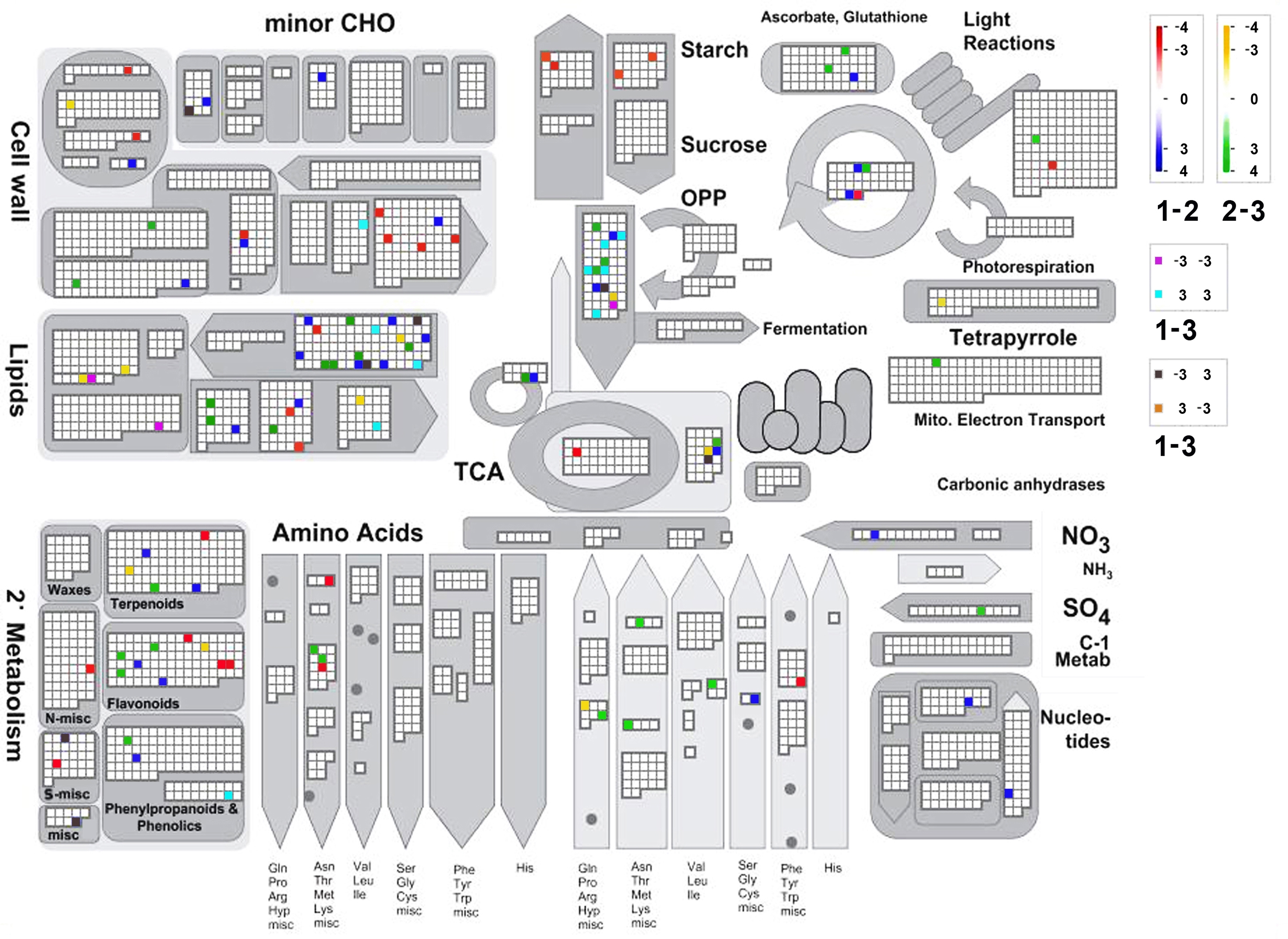

Supplement: Additional file 6 — MAPMAN map. Overview MAPMAN metabolism map showing several constantly and transiently up- and down-regulated genes related to cell wall synthesis and breakdown, fatty acid biosynthesis and lipid breakdown, starch and sucrose metabolism, glycolysis, Calvin cycle and the secondary metabolism (e.g., terpenoids, flavonoids, phenols). Signal colors: blue and green correspond to up-regulated genes between olive stages 1 and 2, and 2 and 3, respectively, whereas red and yellow correspond to down-regulated genes between olive stages 1 and 2, and 2 and 3, respectively; cyan and violet indicate, respectively, constantly up-regulated and down-regulated genes from olive stage 1 to stage 3; gray and orange indicate, respectively, transiently down- and up-regulated genes. [file 1471-2229-9-128-S5.JPEG]
