# Supplementary material for: Computational annotation of genes differentially expressed along olive fruit development
Source: BMC Plant Biol. 2009 Oct 24;9:128. doi: 10.1186/1471-2229-9-128 (PMC2774695; doi:10.1186/1471-2229-9-128)
Supplement: Additional file 2 — List of enzyme names and codes. List of enzyme names, codes and library of the 89 gene sequences tested by quantitative Real-Time PCR. [file 1471-2229-9-128-S7.PDF]

Enzyme Id	Enzyme
ec:1.1.1.1_A	alcohol dehydrogenase
ec:1.1.1.100_B	3-oxoacyl-[acyl-carrier-protein] reductase
ec:1.1.1.219_A	dihydrokaempferol 4-reductase
ec:1.1.1.219_D	dihydrokaempferol 4-reductase
ec:1.1.1.37_C	malate dehydrogenase
ec:1.1.1.37_D	malate dehydrogenase
ec:1.1.1.40_B	malate dehydrogenase (oxaloacetate-decarboxylating) (NADP+)
ec:1.1.1.40_C	malate dehydrogenase (oxaloacetate-decarboxylating) (NADP+)
ec:1.11.1.7_A_1	peroxidase
ec:1.11.1.7_A_2	peroxidase
ec:1.13.11.12_A	lipoxygenase
ec:1.13.11.12_B	lipoxygenase
ec:1.13.11.12_D	lipoxygenase
ec:1.14.11.19_D_1	leucocyanidin oxygenase
ec:1.14.11.19_D_2	leucocyanidin oxygenase
ec:1.14.11.9_D	flavanone 3-dioxygenase
ec:1.14.19.2_D_1	acyl-[acyl-carrier-protein] desaturase
ec:1.14.19.2_D_2	acyl-[acyl-carrier-protein] desaturase
ec:1.15.1.1_D	superoxide dismutase
ec:1.2.1.12_B	glyceraldehyde-3-phosphate dehydrogenase (phosphorylating)
ec:1.2.1.12_B/D	glyceraldehyde-3-phosphate dehydrogenase (phosphorylating)
ec:1.2.1.12_C	glyceraldehyde-3-phosphate dehydrogenase (phosphorylating)
ec:1.3.1.9_C	enoyl-[acyl-carrier-protein] reductase (NADH)
ec:1.6.5.3_D	NADH dehydrogenase (ubiquinone)
ec:1.6.5.4_D	monodehydroascorbate reductase (NADH)
ec:2.2.1.7_A	1-deoxy-D-xylulose-5-phosphate synthase
ec:2.3.1.39_D	[acyl-carrier-protein] S-malonyltransferase
ec:2.3.1.74_D_1	naringenin-chalcone synthase
ec:2.3.1.74_D_2	naringenin-chalcone synthase
ec:2.3.3.8_A_1	ATP citrate synthase / citrate (Si)-synthase
ec:2.3.3.8_A_2	ATP citrate synthase / citrate (Si)-synthase
ec:2.4.1.12_A	cellulose synthase (UDP-forming)
ec:2.4.1.12_B	cellulose synthase (UDP-forming)
ec:2.4.1.241_C	digalactosyldiacylglycerol synthase
ec:2.5.1.18_D	glutathione transferase
ec:2.5.1.6_A_1	methionine adenosyltransferase
ec:2.5.1.6_A_2	methionine adenosyltransferase
ec:2.7.1.11_D	6-phosphofructokinase
ec:2.7.1.20_B	adenosine kinase
ec:2.7.1.33_B	pantothenate kinase
ec:2.7.1.40_B_1	pyruvate kinase
ec:2.7.1.40_B_2	pyruvate kinase
ec:2.7.1.40_B_3	pyruvate kinase
ec:2.7.1.40_C	pyruvate kinase
ec:2.7.11.25_C	mitogen-activated protein kinase

Enzyme Id	Enzyme
ec:2.7.7.27_A	glucose-1-phosphate adenylyltransferase
ec:2.8.1.2_B	3-mercaptopyruvate sulfurtransferase
ec:3.1.1.11_D	pectinesterase
ec:3.1.1.4_A	phospholipase A2
ec:3.1.1.4_D	phospholipase A2
ec:3.1.2.14_A	oleoyl-[acyl-carrier-protein] hydrolase
ec:3.1.3.16_D	phosphoprotein phosphatase
ec:3.1.4.4_A	phospholipase D
ec:3.1.4.4_B	phospholipase D
ec:3.2.1.0_B	alpha-amylase, galactinol
ec:3.2.1.125_C	raucaffricine beta-glucosidase
ec:3.2.1.15_B	polygalacturonase
ec:3.2.1.2_A/B	beta-amylase
ec:3.2.1.21_B	beta-glucosidase
ec:3.2.1.23_D	beta-galactosidase
ec:3.5.1.1_D	asparaginase
ec:3.5.1.4_C	amidase
ec:3.6.3.14_A	H+-transporting two-sector ATPase
ec:4.1.1.19_D	arginine decarboxylase
ec:4.1.1.28_A_1	aromatic-L-amino-acid decarboxylase
ec:4.1.1.28_A_2	aromatic-L-amino-acid decarboxylase
ec:4.1.1.28_A_3	aromatic-L-amino-acid decarboxylase
ec:4.1.1.49_D	phosphoenolpyruvate carboxykinase (ATP)
ec:4.1.1.50_C	adenosylmethionine decarboxylase
ec:4.2.1.11_B_1	phosphopyruvate hydratase
ec:4.2.1.11_B_2	phosphopyruvate hydratase
ec:4.2.1.11_B_3	phosphopyruvate hydratase
ec:4.2.1.11_C	phosphopyruvate hydratase
ec:4.2.2.2_A_1	pectate lyase
ec:4.2.2.2_A_2	pectate lyase
ec:4.2.2.2_A_3	pectate lyase
ec:5.1.3.18_B	GDP-mannose 3,5-epimerase
ec:5.3.1.5_D	xylose isomerase
ec:5.3.3.8_D	dodecenoyl-CoA isomerase
ec:6.3.2.19_D	ubiquitin protein ligase
ec:6.4.1.2_B_1	acetyl-CoA carboxylase
ec:6.4.1.2_B_2	acetyl-CoA carboxylase
ec:6.4.1.2_C	acetyl-CoA carboxylase
hormon_1	senescence-associated protein
hormon_2	aldo/keto reductase family protein
hormon_3	pyridoxine biosynthesis
hormon_4	UDP-glycosyltransferase
hormon_5	arabidopsis response regulator 1
hormon_6	ABA deficient 2
