# Supplementary material for: Computational annotation of genes differentially expressed along olive fruit development
Source: BMC Plant Biol. 2009 Oct 24;9:128. doi: 10.1186/1471-2229-9-128 (PMC2774695; doi:10.1186/1471-2229-9-128)
Supplement: Additional file 5 — Secondary metabolism enzymes. List of enzymes involved in the biosynthesis and metabolism of secondary metabolites, such as steroids, phenylpropanoids, flavonoids, alkaloids and specific products like caffeine, limonene and pinene. [file 1471-2229-9-128-S4.PDF]

Enzyme	Enzyme Id	Library	Biosynthesis of steroids	Caffeine metabolism	Limonene and pinene degradation	Phenylpropanoid biosynthesis	Flavonoid biosynthesis	Alkaloid biosynthesis I and II
Dihydrokaempferol 4-reductase	ec:1.1.1.219	A-D					X	
Peroxidase	ec:1.11.1.7	A-C				X		
Leucocyanidin oxygenase	ec:1.14.11.19	D					X	
Flavanone 3-dioxygenase	ec:1.14.11.9	D					X	
Unspecific monooxygenase	ec:1.14.14.1	A-D		X				
Tetrahydroberberine oxidase	ec:1.3.3.8	D						X
1-deoxy-D-xylulose-5 phosphate synthase	ec:2.2.1.7	A	X					
Naringenin-chalcone synthase	ec:2.3.1.74	D					X	
Carboxylesterase	ec:3.1.1.1	D						X
Beta-glucosidase	ec:3.2.1.21	B				X		
Tyrosine decarboxylase	ec:4.1.1.25	A						X
Aromatic-L-amino-acid decarboxylase	ec:4.1.1.28	A						X
Enoyl-CoA hydratase	ec:4.2.1.17	D			X			
Number of identified enzymes			1	1	1	2	4	4
