# Supplementary material for: Computational annotation of genes differentially expressed along olive fruit development
Source: BMC Plant Biol. 2009 Oct 24;9:128. doi: 10.1186/1471-2229-9-128 (PMC2774695; doi:10.1186/1471-2229-9-128)
Supplement: Additional file 7 — List of specific primers used for RT-PCR analysis. List of specific primers designed on the selected key genes for the validation of their expression patterns by Real-Time PCR analysis. [file 1471-2229-9-128-S6.PDF]

Primer name	Enzyme name	Enzyme Id		Primer sequence
**A_C15_B08**	oleoyl-[acyl-carrier-protein] hydrolase	ec:3.1.2.14_A	Forward	GCTTTCTACTGTCCTCATCTACAAC
			Reverse	GCTTCGAGTCTATGGGTGATG
**A_E21_C11**	methionine adenosyltransferase	ec:2.5.1.6_A_1	Forward	TTTTCACGCTTCAGGTTTCT
			Reverse	CATACGGGCACTTTGGAAG
**A_M18_G09**	alcohol dehydrogenase	ec:1.1.1.1_A	Forward	GCGAATTTTGCAGTCAAGG
			Reverse	TGAAACCACAGCAAAGGAA
**A_M19_G10**	methionine adenosyltransferase	ec:2.5.1.6_A_2	Forward	CCTTCCATTTCCGCCCCT
			Reverse	CCATTGTCTGTGTTTGTTGATACTT
**A_N13_G07**	H+-transporting two-sector ATPase	ec:3.6.3.14_A	Forward	TGACAACAAGGGAAGGGAAAT
			Reverse	GGCAGCAGAGCATCAAGAAT
**A_O01_H01**	phospholipase D	ec:3.1.4.4_A	Forward	GATACTCCATTCCATTCACTTTTC
			Reverse	TGTTTCCTCCATCTCTGCTC
**ADP-glucose**	glucose-1-phosphate adenylyltransferase	ec:2.7.7.27_A	Forward	ATCTGCACCCATCAGCAATGAA
			Reverse	CCTCGATATCTGCCTCCTTCCA
**A-K05-F03**	senescence-associated protein	A-K05-F03_A	Forward	CGTCGTGAGACAGGTTAGTT
			Reverse	GGCGTTCAGTCATAATCCA
**ANTHOCYAN-D-P13**	leucocyanidin oxygenase	ec:1.14.11.19_D_1	Forward	GTGAATAAAGAGAAGGTGAGGA
			Reverse	AGAAAACTGCCCAAGAAATC
**Antocyan_D**	leucocyanidin oxygenase	ec:1.14.11.19_D_2	Forward	GACCCATTTTCCCTCACA
			Reverse	CACTTTCATCCTCCACAACA
**At1g2141_D**	ubiquitin protein ligase	ec:6.3.2.19_D	Forward	CCTCCTTGTATCCCTTTTTCAC
			Reverse	AAACCATTGCCTTCACCTAAGA
**At5g28840_B**	GDP-mannose 3,5-epimerase	ec:5.1.3.18_B	Forward	ACCGATTTGAACTGGAGCTTG
			Reverse	ACGAGATTGAAGGATGGCCTG
**B_B24_A12**	alpha-amylase, galactinol---sucrose galactosyltransferase	ec:3.2.1.0_B	Forward	CAACCCGACCCCCAAGACC
			Reverse	CTGGAATAGATGGCGTGAAGGTT
**B_C08_B04**	pantothenate kinase	ec:2.7.1.33_B	Forward	TCGCAAGAGGGACAGAGATA
			Reverse	ACATACTGGGGATTAGGGAAGC
**B_C12_B06**	adenosine kinase	ec:2.7.1.20_B	Forward	CCTTTCTGCTCCATTTATCTGT
			Reverse	CGTGCTTCCGTTTCATTTCC

**B_G23_D12**	phosphopyruvate hydratase	ec:4.2.1.11_B_1	Forward	CAACCCCACGGAGAGATCAG
			Reverse	CGCAGTGGAGAGACAGAGG
**B_I06_E03**	phospholipase D	ec:3.1.4.4_B	Forward	GCTCGCTCACCTTTCCTTCG
			Reverse	GCAGCCAGCAAGGGGACAAA
**B-A14-A07**	lipoxygenase	ec:1.13.11.12_B	Forward	CTCGGCTACAGACATTACTGG
			Reverse	TCGTTCCTCTACTTCCCTCAA
**BETA-GAL-D-B19**	beta-galactosidase	ec:3.2.1.23_D	Forward	CAACAATGCGATTACAGAG
			Reverse	TCTCCGCCAATCTCTTCA
**B-gluc_B**	pyruvate kinase	ec:2.7.1.40_B_1	Forward	CAGCTGCCAAGAGTGCATCAAT
			Reverse	CAAGGACGATGTTGTCGTGATG
**B-gluc_C**	raucaffricine beta-glucosidase	ec:3.2.1.125_C	Forward	TGGGATATTCCCCAATGTTTGC
			Reverse	CGATCACCAAATTCCCAGAAGC
**Biotin_1B**	acetyl-CoA carboxylase	ec:6.4.1.2_B_1	Forward	CTCCTTCAAATGTCCCTGCTA
			Reverse	TGGGGCAGAAGAACGGTAAAA
**Biotin_3B**	acetyl-CoA carboxylase	ec:6.4.1.2_B_2	Forward	CTCCTTCAAATGTCCCTGCTC
			Reverse	TGGGGCAGAAGAACGGTAAAA
**C_C04_B02**	adenosylmethionine decarboxylase, arginine decarboxylase	ec:4.1.1.50_C	Forward	TCAAATCCAATCGCAGAAAC
			Reverse	TTCCACTTCACTCCTTTCCTT
**C_D14_B07**	digalactosyldiacylglycerol synthase	ec:2.4.1.241_C	Forward	CAGCAGAAAGGCGGAGGA
			Reverse	GGAATACATCAAGAGGGAAAAGA
**C_D19_B10**	mitogen-activated protein kinase kinase kinase	ec:2.7.11.25_C	Forward	GTCTCAAGAGGGAGCCGTTAG
			Reverse	CAAGGGGAGGAGGGAAAGCA
**C_I19_E10**	pyruvate kinase	ec:2.7.1.40_C	Forward	TGATTTTATTGCCTTGTCGTT
			Reverse	GGCTTCCAAACTCTCTATCTT
**C_N01_G01**	amidase	ec:3.5.1.4_C	Forward	AGCCAAACGCAGAATAGAAG
			Reverse	GCAGCAAATCTTCTTGGGTTTC
**Cellulose**	cellulose synthase (UDP-forming)	ec:2.4.1.12_A	Forward	CTGTTTTGCCTTCCCATCAACC
			Reverse	CCCTTCTCATTCCACCAACCAC
**Cellulose_B**	cellulose synthase (UDP-forming)	ec:2.4.1.12_B	Forward	GCAGAATGCAATAAGCGAGCAA
			Reverse	TCAGGTCCTTAGGTGGGCTTT
**C-H22-D11**	aldo/keto reductase family protein	C-H22-D11_C	Forward	GGAAGTGTCGAGGAATGAGA

			Reverse	CTGGGCTGTATGGGTATG
**Chalcone_D**	naringenin-chalcone synthase	ec:2.3.1.74_D_1	Forward	GGCGAAGACCGAGGAGTTT
			Reverse	CCCAAAAAGCAATCAAAGAATGG
**Chloroplast_C**	acetyl-CoA carboxylase	ec:6.4.1.2_C	Forward	AAATGCAGTCTTCTATGTTGCC
			Reverse	GGTTCAGGGATGATGCCG
**Citrate_A**	ATP citrate synthase / citrate (Si)-synthase	ec:2.3.3.8_A_1	Forward	GCAAGACAGATCCAAGATACACG
			Reverse	GTTCTAAGAGTATAGGAGGCACT
**Citrate_B**	ATP citrate synthase / citrate (Si)-synthase	ec:2.3.3.8_A_2	Forward	AAAGCACTTGCCTGAAGATCCA
			Reverse	ACAGTGTAGTATCTTGCTTCAGTC
**C-J08-E04**	pyridoxine biosynthesis	C-J08-E04_C	Forward	TACCCCGAACCCTCTTGT
			Reverse	GTGACGCCTGCTGATGCT
**C-O09-H05**	UDP-glycosyltransferase	C-O09-H05_C	Forward	TTAGCAAGACCCCAAGCAAA
			Reverse	GAGCGAAAACTCAAATCCATT
**Cysteine_A**	phospholipase A2	ec:3.1.1.4_A	Forward	ATGGCTCTATTGCAATACCACA
			Reverse	TGGTGTGGCTGTTGTTGGAT
**Cysteine_D**	phospholipase A2	ec:3.1.1.4_D	Forward	ATGGCTCTATTGCAATACCACG
			Reverse	TGGTGTGGCTGTTGTTGGAT
**D_A03_A02**	asparaginase	ec:3.5.1.1_D	Forward	TCAGGGAGGGGATTGATGAGC
			Reverse	GTGGGTGGCGATGGGTTGG
**D_D01_B01**	superoxide dismutase	ec:1.15.1.1_D	Forward	CTTCCACCAGCATTTCCAGT
			Reverse	CTCTTACAGGACCACATTCCA
**D_D23_B12**	NADH dehydrogenase (ubiquinone)	ec:1.6.5.3_D	Forward	CCACCAAGCACTCATTCTCT
			Reverse	CTCATACCCCGAACCATTTCA
**D_I24_E12**	arginine decarboxylase	ec:4.1.1.19_D	Forward	CAGCCTCACCAACACCATC
			Reverse	GACATTTCGGGTCCACTTC
**D-B23-A12**	naringenin-chalcone synthase	ec:2.3.1.74_D_2	Forward	GGCGAAGACCGAGGAGTTT
			Reverse	CCCAAAAAGCAATCAAAGAATGG
**D-E16-C08**	arabidopsis response regulator 1	D-E16-C08_D	Forward	AGTGACAAACTGCTGATGGAG
			Reverse	GAAAGGACGAGGAAGATGAAG
**Deoxylul_A**	1-deoxy-D-xylulose-5-phosphate synthase	ec:2.2.1.7_A	Forward	GCTGGACAATTGAACCGAATC
			Reverse	TCATAGACGACAGGCCAAG

**Dihydroflav**	dihydrokaempferol 4-reductase	ec:1.1.1.219_D	Forward	GAGCCATGAGCCGATGAAGC
			Reverse	TCTTTTGCGACTCCAATTTCTCG
**Dihydroflav_A**	dihydrokaempferol 4-reductase	ec:1.1.1.219_A	Forward	CTTGTCCAGGGTTCAGGCATCT
			Reverse	GGATGATCTATGTGCGGCTGAA
**D-J04-E02**	ABA deficient 2	D-J04-E02_D	Forward	CCACCATCCACAAAAAGGTTGT
			Reverse	GTTCCTACACGGCAATCAAAG
**D-K14-F07**	lipoxygenase	ec:1.13.11.12_D	Forward	GGAGTCAAGGAACGGTAATG
			Reverse	GAATGAAGCACTGAGTATATTGTT
**Enolase_B**	phosphopyruvate hydratase	ec:4.2.1.11_B_2	Forward	GTTAGCAATGTGCTTGTATAGG
			Reverse	AGGCCGTTAGCAATGTTAACAT
**ENOLASE-C**	phosphopyruvate hydratase	ec:4.2.1.11_C	Forward	GTTAGCAATGTGCTTATATAGC
			Reverse	AGGCCGTTAGCAATGTTAACAC
**Enoyl-acp_C**	enoyl-[acyl-carrier-protein] reductase (NADH)	ec:1.3.1.9_C	Forward	TCAACGGCAGAGTCATTTTCC
			Reverse	CCTCTCAGATCAATGGGCAATC
**Flavanone_D**	flavanone 3-dioxygenase	ec:1.14.11.9_D	Forward	AGCATTCTTGAACCTCCCATTG
			Reverse	GGATGGTGGCAAGACATGGA
**GADPH-BC05**	glyceraldehyde-3-phosphate dehydrogenase (phosphorylating)	ec:1.2.1.12_B	Forward	TCAACCACAGAGACATCAACA
			Reverse	ATCATTCCCAGCAGCACAG
**GADPH-BDCONS**	glyceraldehyde-3-phosphate dehydrogenase (phosphorylating)	ec:1.2.1.12_B/D	Forward	ACCCTCAACAATACCAAACCTG
			Reverse	TCATTTCTGCCCCAAGTAA
**GADPH-CCONS**	glyceraldehyde-3-phosphate dehydrogenase (phosphorylating)	ec:1.2.1.12_C	Forward	CTTCATCAGTGTAGCCGA
			Reverse	TTCCAACCCCTAATGTCTCT
**GLUTAT-ALN179**	glutathione transferase	ec:2.5.1.18_D	Forward	CACACTTTTCCTCTCCTTCAC
			Reverse	CATTTACACTGGCTGATCTGAG
**Lipoxygen_A**	lipoxygenase	ec:1.13.11.12_A	Forward	GACGCTCTTGAATGGACTAAGG
			Reverse	AATTCCCTTGCCAGTAAGTCCC
**MALATE-JO5**	malate dehydrogenase	ec:1.1.1.37_C	Forward	GTAATGAACTCACTATTCAACCAT
			Reverse	TTGGGGAAATCACTCTTCAACG
**MALATE-PO5**	malate dehydrogenase	ec:1.1.1.37_D	Forward	CCATTCATCATCTGCAACAAGT
			Reverse	TTGGGGAAATCACTCTTCAACT
**MALONYL-ACYL-CONS**	[acyl-carrier-protein] S-malonyltransferase	ec:2.3.1.39_D	Forward	ACAATGCCAGCTATAACCTTTCCA

			Reverse	CTCCCAGAATACCAGTTTATC
**MONODEHYD-CONS**	monodehydroascorbate reductase (NADH)	ec:1.6.5.4_D	Forward	GGGAATAGAAGAACGGAAGG
			Reverse	TGAAGAGGAAAAAGGTGGAA
**NADP-malic**	malate dehydrogenase (oxaloacetate-decarboxylating) (NADP+)	ec:1.1.1.40_B	Forward	AGCCCCTTGTTGTGATGTGGAT
			Reverse	GGAGCAAAGAGGTGGGGAGTC
**NADP-MALIC**	malate dehydrogenase (oxaloacetate-decarboxylating) (NADP+)	ec:1.1.1.40_C	Forward	TGCCAAAAATACGGGAGTATC
			Reverse	GCAAGCAGAAGGACGAATAC
**Oxoacylcar_B**	3-oxoacyl-[acyl-carrier-protein] reductase	ec:1.1.1.100_B	Forward	AAACATCCAATGTTCCCCATGC
			Reverse	AAAGGAGGCGGAAGAAGTTT
**Pect_lyase_A**	pectate lyase	ec:4.2.2.2_A_1	Forward	AACACGAGGAGGCACCAGA
			Reverse	CATTGAACCGATAAGAGAAGATG
**Pectin_D**	pectinesterase	ec:3.1.1.11_D	Forward	CGGTGATAATAGTGTTGCTTCT
			Reverse	ATAGGGAAAATGTGGAGGTG
**PECT-LY-ACO1**	pectate lyase	ec:4.2.2.2_A_2	Forward	TGGAAGAAATGGAACTGGAGA
			Reverse	TAAGAGAAGATGGTCTTGCACTC
**PECT-LY-CON11**	pectate lyase	ec:4.2.2.2_A_3	Forward	TGGAAGAAATGGAACTGGAGA
			Reverse	TAAGAGAAGATGGTCTTGCACTT
**PEROXID-CON15-1**	peroxidase	ec:1.11.1.7_A_1	Forward	TTAGCATTTCCTGATTGGTTGT
			Reverse	ATCTCGTGGCATTGTCTGGA
**PEROXID-CON15-2**	peroxidase	ec:1.11.1.7_A_2	Forward	TTAGCATTTCCTGATTGGTTGT
			Reverse	ATCTCGTGGCATTGTCTGGG
**PHOSPHOENOL**	phosphoenolpyruvate carboxykinase (ATP)	ec:4.1.1.49_D	Forward	AACGCAAACTACAAGACAACC
			Reverse	GGCACACCCTCAACCTCA
**PhosPiruv**	phosphopyruvate hydratase	ec:4.2.1.11_B_3	Forward	ACCAAACCCACCTCACAAGCAT
			Reverse	CGTCGGATTGCCTCTGCTATCT
**Protein_B**	polygalacturonase	ec:3.2.1.15_B	Forward	GGGGTCAGGATCAAAACAG
			Reverse	CGCTACCACATCCCTGTA
**PROTEIN-A-B-CONS**	beta-amylase	ec:3.2.1.2_A/B	Forward	GCACACATCTCCCTCTCCTC
			Reverse	AGAGACCACGAGCAACCACA
**Prot-j01_D**	phosphoprotein phosphatase	ec:3.1.3.16_D	Forward	GGTAGGGTTCACAAAAAGTAATC
			Reverse	GAAATCGGGGAAAATATGGAG

**PYROPHOSPH-D**	6-phosphofructokinase	ec:2.7.1.11_D	Forward	TCTCCAATCACATCCCAATATG
			Reverse	CAGCCACCTCTTCAGCAA
**Pyruvate_B1**	beta-glucosidase	ec:3.2.1.21_B	Forward	CAGAAACATCGGCAACTTCAG
			Reverse	CTATGGTGGCAAGAGGAGAT
**Pyruvate_B2**	pyruvate kinase	ec:2.7.1.40_B_2	Forward	GAAGGCCGATAATGGCTCAG
			Reverse	GGTTGGTTCACAGACTCCAC
**PYRUVATE-D-E17**	pyruvate kinase	ec:2.7.1.40_B_3	Forward	TGTGTTATGCTAAGTGGGGAGA
			Reverse	AATCGAATGAATCTGTGGTAT
**STEAROYL-D-B11-A06**	acyl-[acyl-carrier-protein] desaturase	ec:1.14.19.2_D_1	Forward	CAACCCGTCCAGAAAGATAAAG
			Reverse	GCACTTCCCACTTACCAGA
**Steroyl_D**	acyl-[acyl-carrier-protein] desaturase	ec:1.14.19.2_D_2	Forward	GAAGGTGGGATGTTGAGAAGT
			Reverse	CGGATGATGTCTGCTTGA
**Tetrafunct_D**	dodecenoyl-CoA isomerase	ec:5.3.3.8_D	Forward	TTTCGGTTGACATTATTGATA
			Reverse	GTTGATTGGATGCTTTCTATT
**Thiosulfate**	3-mercaptopyruvate sulfurtransferase	ec:2.8.1.2_B	Forward	GTGCAACCCCATCAAACCTAGC
			Reverse	AAATTCCAGCCCCATTTCGTCT
**TYROSINE-A-F15-1**	aromatic-L-amino-acid decarboxylase	ec:4.1.1.28_A_1	Forward	GACCCACATCCCATACTCCTTT
			Reverse	GGCTTGTTCCGCTCTTTCTC
**TYROSINE-CONS**	aromatic-L-amino-acid decarboxylase	ec:4.1.1.28_A_2	Forward	GACCCACATCCCATACTCCTTT
			Reverse	GGCTTGTTCCGCTCTTTCTG
**TYROSINE-A-K15-2**	aromatic-L-amino-acid decarboxylase	ec:4.1.1.28_A_3	Forward	GAGAAGAGAAATGAACTGGGAAGA
			Reverse	TGGGTTTAATGTTGTTGGATTT
**Xylose_D**	xylose isomerase	ec:5.3.1.5_D	Forward	ACTGGCGACCCTCAAACC
			Reverse	AACATCCGTGCTCTCTCTTC
